# Kappa free light chain and neurofilament light independently predict early multiple sclerosis disease activity—a cohort study

**DOI:** 10.1016/j.ebiom.2023.104573

**Published:** 2023-04-20

**Authors:** Harald Hegen, Klaus Berek, Gabriel Bsteh, Michael Auer, Patrick Altmann, Franziska Di Pauli, Astrid Grams, Dejan Milosavljevic, Markus Ponleitner, Paulina Poskaite, Christine Schnabl, Sebastian Wurth, Anne Zinganell, Thomas Berger, Janette Walde, Florian Deisenhammer

**Affiliations:** a Department of Neurology, Medical University of Innsbruck, Innsbruck, Austria; b Department of Neurology, Medical University of Vienna, Vienna, Austria; c Comprehensive Center for Clinical Neurosciences and Mental Health, Medical University of Vienna, Vienna, Austria; d Department of Neuroradiology, Medical University of Innsbruck, Innsbruck, Austria; e FH Campus Wien, University of Applied Sciences, Vienna, Austria; f Department of Neurology, Medical University of Graz, Graz, Austria; g Department of Statistics, Faculty of Economics and Statistics, University of Innsbruck, Innsbruck, Austria

**Keywords:** Cerebrospinal fluid, Kappa free light chain, Neurofilament light, Multiple sclerosis, Disease activity, Prediction

## Abstract

**Background:**

Inter-individual courses of multiple sclerosis (MS) are extremely variable. The objective of this study was to investigate whether κ-free light chain (κ-FLC) index and serum neurofilament light (sNfL) have an additive predictive value for MS disease activity.

**Methods:**

Patients with early MS who had cerebrospinal fluid (CSF) and serum sampling at disease onset were followed for four years. At baseline, age, sex, disease duration, number of T2-hyperintense (T2L), and contrast-enhancing T1 lesions (CEL) on MRI were determined. During follow-up, the occurrence of a second clinical attack and start of disease-modifying treatment (DMT) were registered. κ-FLC was measured by nephelometry, and κ-FLC index calculated as [CSF κ-FLC/serum κ-FLC]/albumin quotient. sNfL was determined by single-molecule array, and age- and body-mass-index adjusted Z scores were calculated.

**Findings:**

A total of 86 patients at a mean age of 33 ± 10 years and with a female predominance of 67% were included; 36 (42%) patients experienced a second clinical attack during follow-up. Cox regression analysis adjusted for age, sex, T2L, CEL, disease and follow-up duration, and DMT use during follow-up revealed that both κ-FLC index as well as sNfL Z score independently predict time to second clinical attack. The chance for freedom of relapse within 12 months was 2% in patients with high levels of κ-FLC index (>100) and high sNfL Z score (>3), 30% in patients with high κ-FLC index (>100) and lower sNfL Z score (≤3), 70% in patients with lower κ-FLC index (≤100) but high sNfL Z score (>3), and 90% in patients with lower levels of κ-FLC index (≤100) and sNfL Z score (≤3).

**Interpretation:**

κ-FLC index and sNfL Z score have an additive predictive value for early MS disease activity that is independent of known predictors.

**Funding:**

This study was funded by a grant of the charitable foundation of the Austrian Multiple Sclerosis Society.

**Research in context**

**Evidence before this study**

κ-Free light chain (κ-FLC) index and serum neurofilament light (sNfL) are both biomarkers that indicate multiple sclerosis (MS) disease activity.

**Added value of this study**

κ-FLC index and sNfL Z score predict time to second clinical attack in patients with early MS not only in addition to known clinical and paraclinical predictors but also independent of each other.

**Implications of all the available evidence**

κ-FLC index and sNfL capture different pathophysiological disease processes and, thus, increase the ability to predict early MS disease activity. The combination of these biomarker might take us one step closer to tailored medicine in MS.

## Introduction

Multiple sclerosis (MS) is a chronic inflammatory immune-mediated disease of the central nervous system (CNS) that mainly affects young adults and bears the risk of physical and cognitive disability.^1^ Inter-individual courses of MS are extremely variable^2^ and weighing benefits versus risks of disease-modifying treatment (DMT) has become one of the main challenges for neurologists counselling patients with MS.^3^ Since criteria guiding treatment decisions are still controversially debated, there is an urgent need of biomarkers to predict disease activity.^3^^,^^4^ So far, the number of brain MRI lesions and the presence of oligoclonal bands (OCB) in the cerebrospinal fluid (CSF) imply some prognostic value and are widely accepted.^5^

In recent years, κ-Free Light Chain (κ-FLC) as well as Neurofilament Light (NfL) have evolved as emerging biomarkers in MS. κ-FLC index which reflects intrathecal B cell activity shows a high diagnostic accuracy in MS,^6^ has significant methodological advantages compared to OCB detection,^7^ and also predicts early MS disease activity independent of demographics, clinical and MRI characteristics.^8^, ^9^, ^10^, ^11^, ^12^ Neurofilament Light (NfL) mirrors axonal damage and also shows a good correlation with MS disease activity already during early course.^13^, ^14^, ^15^, ^16^, ^17^, ^18^ Recent studies have now provided age- and body-mass-index (BMI) adjusted Z scores for serum NfL (sNfL) concentrations.^19^

While the predictive value of κ-FLC index and NfL separately has been reported, there is to date no information whether the combination of κ-FLC index and sNfL, both reflecting different pathophysiological aspects of MS, i.e. inflammation and neuroaxonal damage, show an independent and additive predictive value for early MS disease activity, which was the aim of the present study.

## Methods

### Study design

The design of this study has been described in detail before.^8^ Briefly, patients of the MS clinic of the Department of Neurology, Medical University of Innsbruck, who had a first demyelinating event of the central nervous system, had CSF and serum collection for routine diagnostic purposes at disease onset and received the diagnosis of clinically isolated syndrome (CIS) or relapsing remitting MS according to the McDonald criteria 2017^20^ were included and prospectively followed over a period of 3–4 years.

At baseline, demographic characteristics (sex, age) as well as clinical and paraclinical variables were assessed. Clinical variables comprised disease duration (time between symptom onset and lumbar puncture), type of symptoms and use of corticosteroid treatment. Paraclinical variables were number of hyperintense lesions on T2-weigthed MRI (T2L), number of contrast-enhancing lesions on T1-weighted MRI (CEL), and main CSF findings including OCB status.

During follow-up, the occurrence of a second clinical attack (i.e. conversion to clinically definite MS, CDMS) and start of DMT were registered. Clinical visits were arranged at the treating physician's discretion, usually every three to six months but at least once a year. At each visit, disability status was assessed by the Expanded Disability Status Scale (EDSS).^21^

### Primary endpoint

The endpoint of the study was the time to second clinical attack. A clinical attack was defined as a monophasic clinical episode with patient-reported symptoms and objective findings reflecting a focal or multifocal inflammatory demyelinating event in the CNS, developing acutely or subacutely, with a duration of at least 24 h in the absence of fever or infection.^20^

### κ-FLC assay and calculation of intrathecal FLC synthesis

κ-FLC concentrations in CSF and serum samples were analyzed as part of the previous study^8^ by nephelometry using Behring ProSpec with the serum FLC immunoassay (N Latex FLC kappa assay, Siemens, Erlangen, Germany) according to the manufacturer's instructions. κ-FLC concentrations were detected by latex-conjugated monoclonal antibodies to epitopes that are exposed when κ-FLC circulate freely.^22^^,^^23^

Intrathecal synthesis of κ-FLC was determined as previously published^24^^,^^25^ by following formula considering serum κ-FLC concentrations and blood–CSF–barrier function.

$$\kappa -FLC\;index=\frac{{\kappa -FLC}_{CSF}\;/\;{\kappa -FLC}_{Serum}}{{Albumin}_{CSF}\;/\;{Albumin}_{Serum}}$$

A κ-FLC index >6.1 denoted presence of an intrathecal κ-FLC synthesis (termed as ‘positive’), a κ-FLC index ≤6.1 denoted absence of an intrathecal synthesis (termed as ‘negative’).^6^ A κ-FLC index >100 was considered as ‘high’, a κ-FLC index >6.1 and ≤100 was considered as ‘moderately elevated’.^8^

### NfL assay and calculation of serum Z scores

NfL was measured in CSF and serum using the Simoa Nf-light kit and provided consumables in the Simoa SR-X Analyzer (Quanterix, Lexington, MA, USA).^26^ The NfL assay was performed according to the manufacturer's instructions and protocol, as previously described.^27^ All samples were measured under blinded conditions at the Medical University of Vienna, Department of Neurology.

As sNfL concentrations increase with age and decrease with BMI under physiological conditions, we calculated age- and BMI-adjusted Z scores. This allows to quantify the deviation of each patient's individual sNfL value in comparison to control persons of the same age and BMI, based on a recently published reference database.^19^ Z score >1.5 was defined as elevated (termed as ‘positive’), Z score ≤1.5 was defined as ‘negative’. A Z score >3 was considered as ‘high’, a Z score >1.5 and ≤3 was considered as ‘moderately elevated’.

### Primary research question

Does the combination of κ-FLC index with sNfL Z score in patients with early MS increase the ability to predict the time to second clinical attack?

### Statistical analysis

Statistical analysis was performed using R software.^28^ Distribution of data was assessed by Kolmogorov–Smirnov test and data were displayed as mean ± standard deviation, or as median and interquartile range (IQR). For group comparisons, Mann-Whitney-U test, χ^2^ test or Fisher's test were applied, as appropriate. Spearman correlation coefficient (r) was used for correlation analysis.

To identify predictors of the time to second clinical attack, Cox regression was employed including the independent variables that statistically significantly differed between patients who converted to CDMS and patients who remained stable during follow-up (non-converters), i.e. sex, disease duration, follow-up duration, T2L, CEL (Table 1), as well as the variables of interest, i.e. κ-FLC index, sNfL Z score or CSF NfL. p-values <0.05 were considered statistically significant. Age almost reached the level of statistical significance (p = 0.051) and thus, also due to findings of previous studies,^5^ was included. Additionally, start of DMT was considered, as a potential impact on time to second clinical attack cannot be definitely excluded.

**Table 1:** Demographic, clinical, MRI and CSF characteristics.

	Total	Non-CDMS converter	N	CDMS converter	n	P value
**Baseline**						
Age (years), mean ± SD	33 ± 10	35 ± 11	50	31 ± 8	36	0.051^f^
Sex (female), n (%)	59 (67)	29 (58)	50	30 (83)	36	**0.018**^g^
*Monofocal syndrome*, n (%)	82 (95)	48 (96)	50	34 (94)	36	0.735^g^
Optic neuritis, n (%)^a^	25 (29)	13 (27)	48	12 (35)	34	0.426^g^
Myelitis, n (%)^a^	37 (43)	24 (50)	48	13 (38)	34	0.292^g^
Brainstem/cerebellum, n (%)^a^	19 (22)	10 (21)	48	9 (26)	34	0.551^g^
Other cerebral symptom, n (%)^a^	1 (1)	1 (2)	48	0 (0)	34	0.397^g^
Disease duration (days)^b^	13 (5–38)	18 (7–60)	50	8 (3–21)	36	**0.006**^h^
No corticosteroid treatment before LP, n (%)	55 (70)	34 (77)	44	21 (60)	35	0.357^g^
*Brain MRI*						
Number of T2 hyperintense lesions	10 (3–17)	8 (3–15)	47	10 (7–20)	33	**0.044**^h^
Number of T1 contrast-enhancing lesions	1 (0–2)	0 (0–1)	44	2 (0–3)	27	**0.003**^h^
Dissemination in space, n (%)^c^	58 (73)	31 (66)	47	27 (83)	33	0.135^g^
Dissemination in time, n (%)^c^	39 (55)	19 (43)	44	20 (76)	27	**0.014**^g^
Field strength (1.5 T), n (%)	69 (86)	39 (83)	47	30 (91)	33	0.311^g^
*Cerebrospinal fluid analysis*						
RBC count (/μl)	0 (0–2)	0 (0–8)	48	0 (0–1)	35	0.552^h^
WBC count (/μl)	5 (3–12)	5 (3–12)	48	6 (3–12)	35	0.644^h^
Oligoclonal IgG bands, n (%)	77 (90)	43 (86)	50	34 (94)	36	0.207^g^
CSF κ-FLC (mg/l)	1.91 (0.68–4.99)	1.75 (0.67–3.78)	50	2.79 (0.74–6.67)	36	0.093^h^
Serum κ-FLC (mg/l)	11.60 (9.47–15.50)	11.40 (9.53–15.50)	50	11.75 (9.22–15.35)	36	0.920^h^
CSF NfL (pg/ml)	895 (475–1704)	752 (476–1261)	47	1226 (444–2140)	34	0.177^h^
Serum NfL (pg/ml)	13.8 (8.4–22.7)	12.6 (8.3–18.4)	45	18.7 (9.1–30.4)	33	0.040^h^
Fulfillment of McDonald criteria 2017 at baseline, n (%)	61 (76)	33 (70)	47	28 (85)	33	0.130^g^
**Follow-up**						
Follow-up duration (months)	47 (38–48)	43.6 (35.5–48.0)	50	47.9 (47.0–48.9)	36	**<0.001**^h^
*Disease modifying treatment*						
DMT start before second attack in CDMS converter, or until end of FU in non-CDMS converter, n (%)	20 (23)	11 (22)	50	9 (25)^d^	36	0.745^g^
Time to DMT start (months)	7.0 (3.7–8.4)	7.4 (3.0–11.2)	11	6.9 (3.8–7.9)	9	0.824^h^
Duration of DMT before second attack (months)				9.0 (5.5–12.6)	9	
*Clinical attacks*						
Time to second attack (months)^e^				11.4 (5.1–23.2)	36	
Time to second attack in treated patients (months)^e^				18.0 (13.7–24.3)	9	
Time to second attack in non-treated patients (months)^e^				10.6 (3.1–22.1)	27	
Number of attacks until end of FU	0 (0–2)			2 (1–3)	36	**<0.001**^h^
*Disability*						
EDSS score ≥3.0 at yr 1, n (%)	3 (4)	2 (5)	44	1 (3)	31	0.774^g^
EDSS score ≥3.0 at yr 2, n (%)	6 (9)	2 (6)	36	4 (13)	32	0.314^g^
EDSS score ≥3.0 at yr 3/4 (LCF), n (%)	7 (8)	2 (4)	50	5 (15)	34	0.081^g^

Data are shown as median and interquartile range unless specified otherwise.

CDMS, clinically definite multiple sclerosis; CSF, cerebrospinal fluid; DMT, disease-modifying treatment; EDSS, Expanded Disability Status Scale; FLC, free light chain; FU, follow-up; LCF, last carried forward; LP, lumbar puncture; MRI, magnetic resonance imaging; NfL, Neurofilament Light; Q_alb_, CSF/serum albumin quotient; RBC, red blood cell; SD, standard deviation; WBC, white blood cell.

a Frequencies (%) are shown for patients with monofocal syndrome only.

b Disease duration is the time between disease onset and lumbar puncture.

c Dissemination in space and time was demonstrated by MRI as defined in Thompson et al. Lancet Neurology 2018; 17 (2):162–173.

d DMT administered before the occurrence of a second clinical attack comprised intramuscular Interferon-β-1a (n = 4), glatiramer acetate (n = 2), dimethyl fumarate (n = 1), and consecutively given teriflunomide, dimethyl fumarate (n = 1) as well as glatiramer acetate, dimethyl fumarate (n = 1).

e Time is calculated from disease onset.

f Independent t test was applied.

g Pearson Chi quadrat or Fisher's test were applied.

h Mann Whitney U test was applied.

Non-linearity in relationship between the log-hazard and the covariates was checked with martingale residuals and LOWESS smoother^29^ Existence of influential observations or outliers was examined with a Jackknife procedure, and the proportional hazards assumption was tested by χ^2^ test.^30^ For model quality Cox and Snell's pseudo R^2^ and the concordance were used.

To visualize the effects, we computed the estimated Cox regression survival probabilities separately for each of the possible combinations of negative/positive κ-FLC index and negative/positive sNfL Z scores. The median of these high and low values were used to plug into the estimated Cox regression and to compute the graph. The parameters T2L, CEL, disease duration, and follow-up duration were fixed at their median values, age at the mean value; for the categorical variables no DMT and female sex were used.

For further visualization, we categorized patients according to the extent of biomarker elevation: κ-FLC index ≤100 and sNfL Z score ≤3, κ-FLC index >100 and sNfL Z score ≤3, κ-FLC index ≤100 and sNfL Z score >3, κ-FLC index >100 and sNfL Z score >3. For a finer stratification, we also used 3-level categories, i.e. combined κ-FLC index ≤6.1, 6.1< κ-FLC index ≤100, κ-FLC index >100 each with sNfL Z score ≤1.5, 1.5< sNfL Z score ≤3, sNfL Z score >3.

A post-hoc power analysis for Cox regression with binary and non-binary covariates was computed^31^: the type one error rate was fixed at 5%, the sample size at 86, the standard deviation of the κ-FLC index (sNfL Z score) was set as 50,^1^ the square of the multiple correlation coefficient between the κ-FLC index (sNfL Z score) and the other covariates as 0.2 (no high multiple correlation), proportion of subjects having a second clinical attack as 0.4, the postulated hazard ratio of κ-FLC index (sNfL Z score) as 1.011 from our prior analysis^8^ (1.7, with the assumption that the sNfL Z score has a similar predictive capability as κ-FLC index and considering the different scales of both variables). With these settings a power for κ-FLC index (sNfL Z score) of 89.0% (87.3%) was computed.

Sensitivity analyses were performed regarding to the use of DMT, the type of disease manifestation and the administration of corticosteroids before lumbar puncture. Robustness of findings considering missing values were checked by leaving corresponding co-variates out of the Cox regression.

### Ethics

The study was approved by the ethics committee of the Medical University of Innsbruck (approval number 1244/2019). Written informed consent was obtained from all patients.

### Role of funders

This study was funded by a grant of the charitable foundation of the Austrian Multiple Sclerosis Society. The Funder had no role in study design, data collection, data analyses, interpretation, or writing.

## Results

A total of 86 patients at a mean age of 33 ± 10 years with a female predominance of 67% were included into the study. Most patients showed a monofocal syndrome with myelitis (43%), followed by optic neuritis (29%), brainstem/cerebellar syndrome (22%) or other topography (1%). OCB were positive in 90% of patients. During follow-up of median 47 months, 36 (42%) of 86 patients converted to CDMS. Twenty (23%) of 86 patients received early DMT, between disease onset and the date of conversion to CDMS for converters, or during follow-up for non-converters. Of 84 patients with available EDSS data at follow-up, 7 (8%) reached an EDSS score of 3.0. Detailed demographic and clinical characteristics, CSF and MRI findings are displayed in Table 1.

### κ-FLC index and sNfL Z score are increased in patients who convert to clinically definite multiple sclerosis

At baseline, κ-FLC index had a median of 36.5 (IQR 15.8–83.1) and was denoted positive in 76 (88%) of 86 patients. sNfL showed a median concentration of 13.8 pg/ml (IQR 8.4–22.7); the median age- and BMI-adjusted sNfL Z score was 2.06 (IQR 0.95–2.87) and was considered positive in 52 patients. CSF NfL had a median of 895 pg/ml (IQR 475–1704).

κ-FLC index, sNfL, sNfL Z score and CSF NfL showed a correlation with CEL and T2L on MRI as shown in Fig. e-1 and Fig. e-2. κ-FLC index statistically significantly correlated with CSF white blood cell (WBC) count (r = 0.53, p < 0.001), while NfL levels did not (sNfL: r = −0.04, sNfL Z score: r = −0.005; CSF NfL: r = 0.20; each p > 0.05). Correlation between CSF NfL and sNfL is shown in Fig. e-3. There was no correlation between κ-FLC index and NfL levels (sNfL: r = −0.05, sNfL Z score: r = −0.05; CSF NfL: r = −0.0003; each p > 0.05). Correlation between κ-FLC index and sNfL Z score is shown in Fig. e-4.

**Fig. 1:**
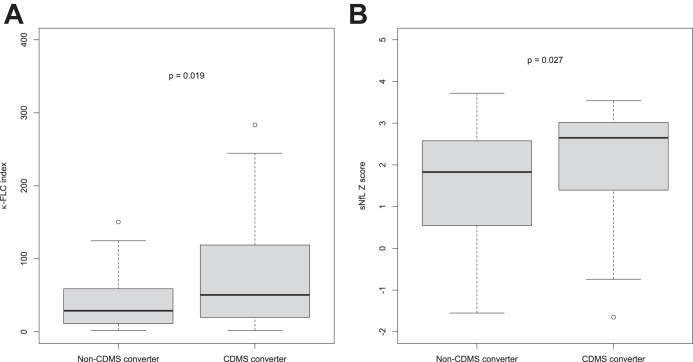
Increased κ-FLC index and sNfL Z score in patients who convert to CDMS. **(A)** κ-FLC index at baseline is significantly higher in patients who convert to CDMS (n = 36) during 4-year follow-up compared to patients who remain relapse-free (n = 50). **(B)** sNfL Z score at baseline is significantly higher in patients who convert to CDMS (n = 33) during 4-year follow-up compared to patients who remain relapse-free (n = 45). Mann–Whitney U test was used for group comparison. CDMS, clinically definite MS; κ-FLC, κ free light chain; sNfL, serum neurofilament light.

κ-FLC index and sNfL Z score were statistically significantly elevated in patients who converted to CDMS during follow-up as compared to non-converters (Fig. 1). For absolute sNfL and CSF NfL concentration we refer to Fig. e-5.

### High κ-FLC index and high sNfL Z score are associated with shorter time to clinically definite multiple sclerosis

**Fig. 2:**
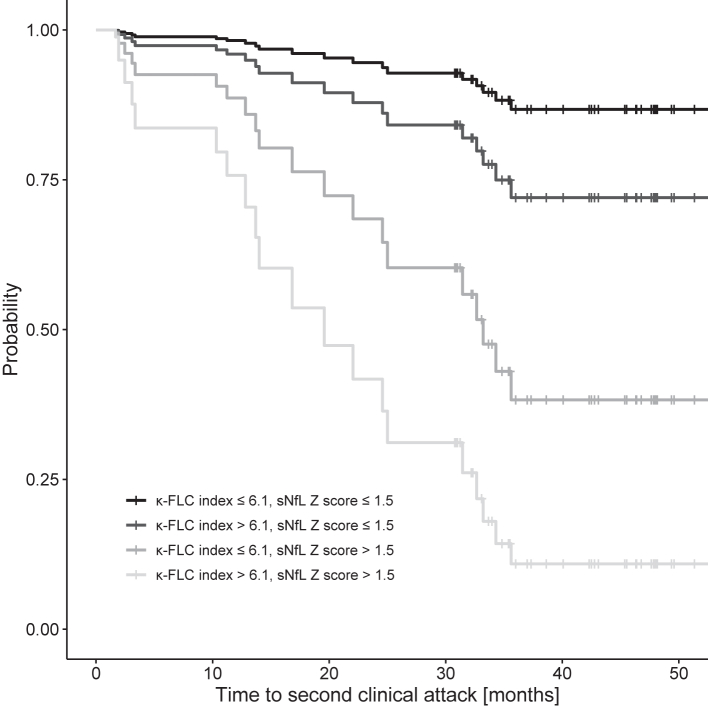
Probability of clinically definite multiple sclerosis over 4 years. The probability of developing a second clinical attack during the 4-year follow-up period is shown separately for each of the possible combinations of positive/negative κ-FLC index and sNfL Z score. κ-FLC, κ free light chain; sNfL, serum neurofilament light.

To investigate whether κ-FLC index and sNfL Z score predict the time to CDMS conversion, multivariable Cox regression model including age, sex, number of T2L, number of CEL, disease duration and follow-up duration as well as the administration of DMT during follow-up was performed. Both, κ-FLC index and sNfL Z score, were independent risk factors for the time to CDMS conversion (Table 2). For univariate analyses see Table e-1. κ-FLC index had a hazard ratio (HR) of 1.23 per increase of κ-FLC index by 10 (p < 0.001), i.e. an increase of κ-FLC index by 10 means a 23% higher risk for conversion to CDMS. sNfL Z score had a HR of 1.08 for an increase in the Z score by 0.1 (p = 0.003), i.e. an increase of the sNfL Z score by 0.1 indicates an 8% higher risk for CDMS conversion (Table 2). The probability for a second clinical attack and freedom thereof showed a stepwise increase depending on whether one or both biomarkers were positive (Fig. 2).

**Table 2:** Cox regression analysis identifiying κ-FLC index and sNfL Z score as predictors for time to second clinical attack.

	Coefficient	Standard error	Hazard ratio	95%-CI	P value
Age (years)	−0.051	0.032	0.950	0.892–1.012	0.109
Sex (ref: male)	0.462	0.650	1.587	0.443–5.677	0.478
Disease duration (days)	−0.024	0.010	0.976	0.957–0.996	**0.018**
Follow-up duration (months)	0.204	0.079	1.226	1.050–1.433	**0.010**
Number of T2 hyperintense lesions	−0.021	0.013	0.979	0.955–1.004	0.099
Number of T1 contrast-enhancing lesions	−0.096	0.219	0.909	0.592–1.395	0.661
DMT administration	0.427	0.732	1.532	0.365–6.438	0.560
κ-FLC index	0.021	0.006	1.021	1.010–1.032	**<0.001**
sNfL Z score	0.784	0.268	2.191	1.300–3.706	**0.003**

Cox and Snell's pseudo R^2^ = 0.481.

Concordance = 0.834 (SE = 0.047).

Disease duration was the time between symptom onset and lumbar puncture. Age was determined at the time of lumbar puncture. Number of MRI lesions were also determined at baseline. Follow-up duration was the time between disease onset and the last clinical visit. DMT administration was determined until occurrence of second clinical attack or end of follow-up, respectively.

Model quality: Covariates were properly included (no necessity for non-linearity). No influential observations were detected (Jackknife procedure revealed that after excluding each patient once all estimates were within the 95%-CI of the estimates of original patient cohort). Proportional hazards assumption was met (χ^2^ = 11.267, p = 0.258).

CI, confidence interval; DMT, disease-modifying treatment; FLC, free light chain; MRI, magnetic resonance imaging; sNfL, serum neurofilament light.

Stratification of patients by the extent of biomarker elevation further showed a stepwise relapse probability. The chance for freedom of relapse within 12 months was 2% in patients with high levels of κ-FLC index (>100) and high sNfL Z score (>3), 30% in patients with high κ-FLC index (>100) and lower sNfL Z score (≤3), 70% in patients with lower κ-FLC index (≤100) but high sNfL Z score (>3), and 90% in patients with lower levels of κ-FLC index (≤100) and sNfL Z score (≤3) (Table e−2). Estimated median time to second clinical attack was 25 months in patients with low κ-FLC index but high sNfL Z score, 17 months in patients with high κ-FLC index but low sNfL Z score, and 3 months in those with high levels of both biomarkers. For a finer stratification of probabilities for freedom of relapse, we refer to Table 3.

**Table 3:** Relapse free probability at 12 months depending on κ-FLC index and sNfL Z score estimated by Cox regression.




The probability (and 95%-confidence interval) of staying relapse-free within 12 months after disease onset is given for each of the possible combinations of negative, elevated and highly elevated κ-FLC index and sNfL Z score.

Number of clinical attacks and patients per category are given from left to right and top to bottom: 0/3, 1/5, 0/2, 4/13, 11/31, 5/7, 4/8, 3/4, 3/3.

κ-FLC, κ free light chain; sNfL, serum neurofilament light.

The distribution of κ-FLC index and sNfL Z score values and the absolute number of patients within negative, positive, low and high biomarker categories are shown in Fig. e-6.

Various sensitivity analyses support the robustness of our findings. Cox regression analyses without DMT (Table e-3), or considering also the type of disease manifestation (Table e-4), or the administration of corticosteroids before lumbar puncture (Table e-5), or storage time (Table e-6) as additional independent control variable consistently showed an effect of κ-FLC index and sNfL z score (with similar estimated coefficients). Cox regression including only a priori known variables as well as relapse free probabilities based on this model are given in Table e-7. As supplement, the results of the Cox regression including CSF NfL (together with κ-FLC index) are shown in Table e-8.

## Discussion

In this study, we demonstrated that the combination of κ-FLC index and sNfL Z score determined at the time of diagnostic lumbar puncture in patients with a first CNS demyelinating event predict the time to a second clinical attack, that is conversion to CDMS, independent of other prognostic factors including load and activity of brain MRI lesions as well as independent of administered DMT. For the first time, we showed that the prognostic values of these two biomarkers are not only additive to known clinical and paraclinical predictors but also independent of each other.

κ-FLC index and sNfL Z score reflect the two main pathophysiological processes of MS, i.e. inflammation and neuroaxonal damage, both of which occur already in the very early disease course.^32^ κ-FLC are intrathecally produced in approximately 90% of MS patients.^6^ We and others have shown that κ-FLC index correlates with MRI activity and CSF white blood cell count at disease manifestation and that κ-FLC index is prognostic for conversion to CDMS.^7^, ^8^, ^9^ Similarly, several studies reported a correlation of sNfL with MRI activity at baseline^15^^,^^33^ as well as a prognostic value for conversion to CDMS during follow-up.^13^^,^^19^ In contrast to κ-FLC index, a correlation of sNfL with CSF white blood cell count was not observed.

This indicates that each of the two biomarkers shows a similar pattern of association with other baseline co-variables and clinical endpoints; however, we did not find a correlation between sNfL Z score and κ-FLC index (r = −0.05). This is of interest, as one might hypothesize that if κ-FLC index correlates e.g. with MRI CEL and MRI CEL with sNfL Z score, κ-FLC index should also correlate with sNfL Z score. As this is obviously not the case in our cohort, one might speculate that κ-FLC index and sNfL Z score reflect—at least to some extent—different patient (sub) groups and/or different aspects of pathophysiology. Recent studies made similar observations reporting an independent predictive value of OCB and sNfL concentration.^13^^,^^34^ Further studies including higher number of patients are needed to investigate how patients differ between a more inflammatory (i.e. high κ-FLC index, low sNfL Z score) and a more ‘destructive’ (i.e. low κ-FLC index, high sNfL Z score) type in terms of clinical and paraclinical characteristics.

In clinical practice, improvement of risk stratification in early MS is of high importance. By using a multivariable analysis, we identified the independent prognostic effect of κ-FLC index and sNfL Z score weighing their impact on the outcome in comparison with the remaining baseline characteristics representing the available arsenal of clinical and paraclinical predictors. Using this representative cohort of patients with a first demyelinating CNS event, we showed that the combination of both biomarkers led to a powerful risk stratification. While elevation of either κ-FLC index (>100) or sNfL Z score (>3) showed only a probability of approximately 20–30% for further relapse within 12 months, the additive effect of both biomarkers allowed the identification of high risk patients. Patients with elevated κ-FLC index and sNfL Z score had a risk of 98% for relapse within 12 months, while patients with negative levels of both biomarkers had a risk of less than 5%. Such a reliable identification of patients at risk for early MS disease activity would have a high impact on daily clinical routine. Patients at high risk could be advised to start DMT early and even use highly effective DMT. There is evidence that the time to the second attack has a prognostic impact on long-term disability^35^^,^^36^ and that early treatment not only delays second clinical attack but much more importantly disability progression.^37^, ^38^, ^39^, ^40^, ^41^, ^42^, ^43^, ^44^ Conversely, there is a certain proportion of patients who shows a mild disease course, probably identified by both low or normal κ-FLC index and sNfL Z score, who may not need a potentially harmful, psychologically distressing and, last but not least, costly DMT.

Interestingly, disease duration differed between patients who converted to CDMS and non-converters (median 8 versus 18 days). One might hypothesize that shorter disease duration, i.e. shorter time period between symptom onset and CSF/serum sample withdrawal, might be linked to severity of onset symptoms. And, the latter might be another surrogate for (further) disease activity. However, as we did not have the information on onset severity, this consideration remains speculative. Including severity of symptoms as well as the degree of remission might be informative co-variables in future studies on κ-FLC index and sNfL Z score.

There are some limitations to this study. First, not all CSF and serum samples were collected before administration of corticosteroids. Although it has been recently shown that high-dose corticosteroids did not affect κ-FLC index,^45^ evidence on sNfL is still lacking. However, in the present study, the proportion of patients with corticosteroid treatment before lumbar puncture did not differ between patients who converted to CDMS and non-converters. Corticosteroid treatment as independent variable as well as its interaction effect with κ-FLC index and sNfL Z score in the regression analysis did not reveal any impact on the time to CDMS conversion nor on both biomarker estimates. The κ-FLC index and sNfL Z score were comparable between the groups (Fig. e-7). Nevertheless, it has to be stated that an effect of corticosteroids cannot be ultimately excluded due to the limited number of patients. Secondly, we measured κ-FLC and NfL concentrations out of frozen and thawed CSF and serum samples after medium-term storage at −80 °C. However, no relevant effect of freezing has been reported for κ-FLC^46^ as well as for NfL.^47^ Also, adding storage time to the multivariable Cox regression model did not change the overall result (Table e-6). In our study, we used the time to second clinical attack as endpoint, and a clinical attack had to be confirmed by a physician. We are aware of the fact that non-confirmed clinical attacks might have occurred,^48^ which means that results could be biased. Further studies on the two biomarkers for prediction of MS disease activity should consider different definitions of clinical attacks. At this point, we also have to state that although we achieved statistically significant hazard ratios for both the κ-FLC index and sNfL Z score, the number of patients and the number of events (i.e. clinical attacks during follow-up) were small. This means that results that are not statistically significant (e.g. MRI parameters) might still have an impact.

This study provides evidence that κ-FLC index and sNfL Z score are additive prognostic biomarkers in MS that capture different pathophysiological processes and might take us one step closer to tailored medicine in MS. Further studies in a multicenter setting including a higher number of patients are required to replicate the additive prognostic value of κ-FLC index and sNfL Z score.

## Contributors

H. Hegen has participated in the conception and design of the study, acquisition of the data, statistical analysis of the data, and in drafting the manuscript.

K. Berek has participated in acquisition of the data and reviewing the manuscript for intellectual content.

G. Bsteh has participated in acquisition of the data and reviewing the manuscript for intellectual content.

M. Auer has participated in reviewing the manuscript for intellectual content.

P. Altmann has participated in reviewing the manuscript for intellectual content.

F. Di Pauli has participated in reviewing the manuscript for intellectual content.

A. Grams has participated in acquisition of the data and reviewing the manuscript for intellectual content.

D. Milosavljevic has participated in acquisition of the data and reviewing the manuscript for intellectual content.

M. Ponleitner has participated in reviewing the manuscript for intellectual content.

P. Poskaite has participated in acquisition of the data and reviewing the manuscript for intellectual content.

C. Schnabl has participated in reviewing the manuscript for intellectual content.

S. Wurth has participated in reviewing the manuscript for intellectual content.

A. Zinganell has participated in reviewing the manuscript for intellectual content.

T. Berger has participated in reviewing the manuscript for intellectual content.

J. Walde has participated in statistical analysis of the data and reviewing the manuscript for intellectual content.

F. Deisenhammer has participated in reviewing the manuscript for intellectual content.

All authors read and approved the final version of the manuscript. HH, KB and JW have accessed and verified all data.

## Data sharing statement

Anonymized data will be shared upon reasonable request from any qualified investigator, i.e. after approval of a proposal and with a signed data access agreement.

## Declaration of interests

HH has participated in meetings sponsored by, received speaker honoraria or travel funding from Bayer, Biogen, Celgene, Merck, Novartis, Sanofi-Genzyme, Siemens, Teva, and received honoraria for acting as consultant for Biogen, Celgene, Novartis and Teva. He is associate editor of Frontiers in Neurology.

KB has participated in meetings sponsored by and received travel funding or speaker honoraria from Roche, Teva, Merck, Biogen, Sanofi.

GB has participated in meetings sponsored by, received speaker honoraria or travel funding from Biogen, Celgene, Lilly, Merck, Novartis, Sanofi-Genzyme and Teva, and received honoraria for consulting Biogen, Celgene, Merck, Novartis, Roche and Teva.

MA received speaker honoraria and/or travel grants from Biogen, Novartis, Merck and Sanofi.

PA has participated in meetings sponsored by, received speaker honoraria or travel funding from Biogen, Merck, Roche, Sanofi-Genzyme and Teva, and received honoraria for consulting from Biogen. He received a research grant from Quanterix International and was awarded a combined sponsorship from Biogen, Merck, Sanofi-Genzyme, Roche, and Teva for a clinical study.

FDP has participated in meetings sponsored by, received honoraria (lectures, advisory boards, consultations) or travel funding from Bayer, Biogen, Celgene, Merck, Novartis, Sanofi-Genzyme, Roche and Teva.

AG has nothing to disclose.

DM has participated in meetings sponsored by Siemens.

MP has participated in meetings sponsored by, received speaker or consulting honoraria or travel funding from Amicus, Merck, Novartis and Sanofi-Genzyme.

PP has nothing to disclose.

CS has participated in meetings sponsored by Siemens.

SW has participated in meetings sponsored by, received honoraria or travel funding from Allergan, Biogen, Ipsen Pharma, Merck, Novartis, Roche, Sanofi Genzyme, Teva and Bristol Myers Squibb.

AZ has participated in meetings sponsored by, received speaking honoraria or travel funding from Biogen, Merck, Novartis, Sanofi-Genzyme and Teva.

TB has participated in the last 2 years in meetings sponsored by and received honoraria (lectures, advisory boards, consultations) from pharmaceutical companies marketing treatments for multiple sclerosis: Almirall, Biogen, Bionorica, BMS/Celgene, Eisai, Horizon, Jazz Pharmaceuticals, Janssen-Cilag, MedDay, Merck, Novartis, Roche, Sanofi Aventis/Genzyme, Sandoz, TG Therapeutics, TEVA and UCB. His institution has received financial support in the last 2 years by unrestricted research grants (Biogen, BMS/Celgene, Novartis, Sanofi Aventis/Genzyme, Roche, TEVA) and for participation in clinical trials in multiple sclerosis sponsored by Alexion, Bayer, Biogen, BMS/Celegen, Merck, Novartis, Roche, Sanofi Aventis/Genzyme, TEVA.

JW has nothing to disclose.

FD has participated in meetings sponsored by or received honoraria for acting as an advisor/speaker for Alexion, Almirall, Biogen, Celgene-BMS, Genzyme-Sanofi, Horizon, Merck, Novartis Pharma, Roche, and Teva. His institution has received research grants from Biogen and Genzyme Sanofi. He is section editor of the MSARD Journal (Multiple Sclerosis and Related Disorders) and review editor of Frontiers Neurology.

## Appendix A Supplementary data

Supplementary Tables e-1–e-8

Supplementary Fig. e-1

Supplementary Fig. e-2

Supplementary Fig. e-3

Supplementary Fig. e-4

Supplementary Fig. e-5

Supplementary Fig. e-6

Supplementary Fig. e-7

Supplementary Fig. e-8

Supplementary figure captions

## References

[1] Compston A., Coles A. (2008). Multiple sclerosis. Lancet.

[2] Weinshenker B.G., Bass B., Rice G.P. (1989). The natural history of multiple sclerosis: a geographically based study. I. Clinical course and disability. Brain.

[3] Ontaneda D., Tallantyre E., Kalincik T., Planchon S.M., Evangelou N. (2019). Early highly effective versus escalation treatment approaches in relapsing multiple sclerosis. Lancet Neurol.

[4] Bsteh G., Hegen H., Dosser C. (2019). To treat or not to treat: sequential individualized treatment evaluation in relapsing multiple sclerosis. Mult Scler Relat Disord.

[5] Tintore M., Rovira À., Río J. (2015). Defining high, medium and low impact prognostic factors for developing multiple sclerosis. Brain.

[6] Hegen H., Walde J., Berek K. (2023). Cerebrospinal fluid kappa free light chains for the diagnosis of multiple sclerosis: a systematic review and meta-analysis. Mult Scler.

[7] Hegen H., Berek K., Deisenhammer F. (2022). Cerebrospinal fluid kappa free light chains as biomarker in multiple sclerosis-from diagnosis to prediction of disease activity. Wien Med Wochenschr.

[8] Berek K., Bsteh G., Auer M. (2021). Kappa-free light chains in CSF predict early multiple sclerosis disease activity. Neurol Neuroimmunol Neuroinflamm.

[9] Arrambide G., Espejo C., Carbonell-Mirabent P. (2022). The kappa free light chain index and oligoclonal bands have a similar role in the McDonald criteria. Brain.

[10] Menéndez-Valladares P., García-Sánchez M.I., Cuadri Benítez P. (2015). Free kappa light chains in cerebrospinal fluid as a biomarker to assess risk conversion to multiple sclerosis. Mult Scler J Exp Transl Clin.

[11] Gaetani L., Di Carlo M., Brachelente G. (2020). Cerebrospinal fluid free light chains compared to oligoclonal bands as biomarkers in multiple sclerosis. J Neuroimmunol.

[12] Salavisa M., Paixão P., Ladeira A.F. (2020). Prognostic value of kappa free light chains determination in first-ever multiple sclerosis relapse. J Neuroimmunol.

[13] Dalla Costa G., Martinelli V., Sangalli F. (2019). Prognostic value of serum neurofilaments in patients with clinically isolated syndromes. Neurology.

[14] Barro C., Benkert P., Disanto G. (2018). Serum neurofilament as a predictor of disease worsening and brain and spinal cord atrophy in multiple sclerosis. Brain.

[15] Disanto G., Barro C., Benkert P. (2017). Serum Neurofilament light: a biomarker of neuronal damage in multiple sclerosis. Ann Neurol.

[16] Lin T.Y., Vitkova V., Asseyer S. (2021). Increased serum neurofilament light and thin ganglion cell-inner plexiform layer are additive risk factors for disease activity in early multiple sclerosis. Neurol Neuroimmunol Neuroinflamm.

[17] Sellebjerg F., Royen L., Soelberg Sørensen P., Oturai A.B., Jensen P.E.H. (2019). Prognostic value of cerebrospinal fluid neurofilament light chain and chitinase-3-like-1 in newly diagnosed patients with multiple sclerosis. Mult Scler.

[18] Håkansson I., Tisell A., Cassel P. (2017). Neurofilament light chain in cerebrospinal fluid and prediction of disease activity in clinically isolated syndrome and relapsing-remitting multiple sclerosis. Eur J Neurol.

[19] Benkert P., Meier S., Schaedelin S. (2022). Serum neurofilament light chain for individual prognostication of disease activity in people with multiple sclerosis: a retrospective modelling and validation study. Lancet Neurol.

[20] Thompson A.J., Banwell B.L., Barkhof F. (2018). Diagnosis of multiple sclerosis: 2017 revisions of the McDonald criteria. Lancet Neurol.

[21] Kurtzke J.F. (1983). Rating neurologic impairment in multiple sclerosis: an expanded disability status scale (EDSS). Neurology.

[22] Hoedemakers R.M., Pruijt J.F., Hol S. (2011). Clinical comparison of new monoclonal antibody-based nephelometric assays for free light chain kappa and lambda to polyclonal antibody-based assays and immunofixation electrophoresis. Clin Chem Lab Med.

[23] Velthuis H.T., Knop I., Stam P. (2011). N Latex FLC - new monoclonal high-performance assays for the determination of free light chain kappa and lambda. Clin Chem Lab Med.

[24] Presslauer S., Milosavljevic D., Huebl W. (2016). Validation of kappa free light chains as a diagnostic biomarker in multiple sclerosis and clinically isolated syndrome: a multicenter study. Mult Scler.

[25] Leurs C.E., Twaalfhoven H., Lissenberg-Witte B.I. (2020). Kappa free light chains is a valid tool in the diagnostics of MS: a large multicenter study. Mult Scler.

[26] Rissin D.M., Kan C.W., Campbell T.G. (2010). Single-molecule enzyme-linked immunosorbent assay detects serum proteins at subfemtomolar concentrations. Nat Biotechnol.

[27] Quanterix Data sheets and technical notes for NF-light. https://www.quanterix.com/products-technology/assays/nf-lightr-sr-x-version.

[28] Core Team R (2018). http://www.R-project.org.

[29] Cleveland W.S. (1981). LOWESS: a program for smoothing scatterplots by robust locally weighted regression. Am Statistician.

[30] Grambsch P.M., Therneau T.M. (1994). Proportional hazards tests and diagnostics based on weighted residuals. Biometrika.

[31] Hsieh F., Lavori P.W. (2000). Sample-size calculations for the Cox proportional hazards regression model with nonbinary covariates. Control Clin Trials.

[32] Trapp B.D., Peterson J., Ransohoff R.M., Rudick R., Mörk S., Bö L. (1998). Axonal transection in the lesions of multiple sclerosis. N Engl J Med.

[33] Disanto G., Adiutori R., Dobson R. (2016). Serum neurofilament light chain levels are increased in patients with a clinically isolated syndrome. J Neurol Neurosurg Psychiatry.

[34] Bittner S., Steffen F., Uphaus T. (2020). Clinical implications of serum neurofilament in newly diagnosed MS patients: a longitudinal multicentre cohort study. eBioMedicine.

[35] Scalfari A., Neuhaus A., Degenhardt A. (2010). The natural history of multiple sclerosis: a geographically based study 10: relapses and long-term disability. Brain.

[36] Tremlett H., Yousefi M., Devonshire V., Rieckmann P., Zhao Y. (2009). Impact of multiple sclerosis relapses on progression diminishes with time. Neurology.

[37] Hauser S.L., Bar-Or A., Cohen J.A. (2020). Ofatumumab versus teriflunomide in multiple sclerosis. N Engl J Med.

[38] Hauser S.L., Bar-Or A., Comi G. (2017). Ocrelizumab versus interferon beta-1a in relapsing multiple sclerosis. N Engl J Med.

[39] Calabresi P.A., Radue E.W., Goodin D. (2014). Safety and efficacy of fingolimod in patients with relapsing-remitting multiple sclerosis (FREEDOMS II): a double-blind, randomised, placebo-controlled, phase 3 trial. Lancet Neurol.

[40] Gold R., Kappos L., Arnold D.L. (2012). Placebo-controlled phase 3 study of oral BG-12 for relapsing multiple sclerosis. N Engl J Med.

[41] Kappos L., Polman C.H., Freedman M.S. (2006). Treatment with interferon beta-1b delays conversion to clinically definite and McDonald MS in patients with clinically isolated syndromes. Neurology.

[42] Comi G., Filippi M., Barkhof F. (2001). Effect of early interferon treatment on conversion to definite multiple sclerosis: a randomised study. Lancet.

[43] Jacobs L.D., Beck R.W., Simon J.H. (2000). Intramuscular interferon beta-1a therapy initiated during a first demyelinating event in multiple sclerosis. CHAMPS Study Group. N Engl J Med.

[44] Polman C.H., O'Connor P.W., Havrdova E. (2006). A randomized, placebo-controlled trial of natalizumab for relapsing multiple sclerosis. N Engl J Med.

[45] Konen F.F., Wurster U., Witte T. (2020). The impact of immunomodulatory treatment on kappa free light chains as biomarker in neuroinflammation. Cells.

[46] Hörber S., Klein R., Peter A. (2019). Effects of long-term storage on serum free light chain stability. Clin Lab.

[47] Altmann P., Leutmezer F., Zach H. (2020). Serum neurofilament light chain withstands delayed freezing and repeated thawing. Sci Rep.

[48] Sorensen P.S., Lycke J., Erälinna J.P. (2011). Simvastatin as add-on therapy to interferon β-1a for relapsing-remitting multiple sclerosis (SIMCOMBIN study): a placebo-controlled randomised phase 4 trial. Lancet Neurol.

