# Supplementary material for: Kappa free light chain and neurofilament light independently predict early multiple sclerosis disease activity—a cohort study
Source: eBioMedicine. 2023 Apr 20;91:104573. doi: 10.1016/j.ebiom.2023.104573 (PMC10148088; doi:10.1016/j.ebiom.2023.104573)
Supplement: Supplementary Tables e-1–e-8 [file mmc1.docx]

**Table e-1:** Univariate Cox regression analyses for each variable separately to predict time to second clinical attack

|  | **Hazard ratio** | **95%-CI** | **P value** |
| --- | --- | --- | --- |
| Age (years) | 0.963 | 0.930 – 0.997 | **0.036** |
| Sex (ref: male) | 2.345 | 1.026 – 5.360 | **0.043** |
| Disease duration (days) | 0.989 | 0.979 – 1.000 | 0.061 |
| Follow-up duration (months) | 1.109 | 1.031 – 1.193 | **0.006** |
| Number of T2 hyperintense lesions | 1.016 | 1.000 – 1.032 | **0.045** |
| Number of T1 contrast-enhancing lesions | 1.199 | 0.980 – 1.466 | 0.077 |
| DMT administration | 0.882 | 0.402 – 1.937 | 0.755 |
| κ-FLC index | 1.009 | 1.004 – 1.015 | **<0.001** |
| sNfL Z score | 1.350 | 0.991 – 1.839 | 0.057 |

Legend:

Disease duration was the time between symptom onset and lumbar puncture. Age was determined at the time of lumbar puncture. Number of MRI lesions were also determined at baseline. Follow-up duration was the time between disease onset and the last clinical visit. DMT administration was determined until occurrence of second clinical attack or end of follow-up, respectively.

*Abbreviations*: CI, confidence interval; DMT, disease-modifying treatment; FLC, free light chain; MRI, magnetic resonance imaging; sNfL, serum neurofilament light

**Table e-2**: Relapse free probability at 12 months depending on κ-FLC index and sNfL Z score estimated by Cox regression

|  | | **sNfL Z score** | |
| --- | --- | --- | --- |
|  |  | $\leq$**3** | $>$**3** |
| **κ-FLC index** | $\leq$**100** | **90%**  (81-100) | **70%**  (52-95) |
|  | $>$**100** | **30%**  (20-44) | **1.8%**  (1.6-2.0) |

Legend:

The probability (and 95%-confidence interval) of staying relapse-free within 12 months after disease onset is given for each of the possible combinations of low and high κ-FLC index and sNfL Z score.

Number of clinical attacks and patients per category are given from left to right and top to bottom: 16/52, 5/9, 7/12, 3/3.

*Abbreviations*: κ-FLC, κ free light chain; sNfL, serum neurofilament light

**Table e-3**: Cox regression analysis without DMT as co-variable to identify predictors for time to second clinical attack

|  | **Coefficient** | **Hazard ratio** | **Standard error** | **P value** |
| --- | --- | --- | --- | --- |
| Age (years) | -0.0568 | 0.9448 | 0.0315 | 0.072 |
| Sex (ref: male) | 0.3416 | 1.4073 | 0.6055 | 0.573 |
| Disease duration (days) | -0.0237 | 0.9766 | 0.0099 | **0.017** |
| Follow-up duration (months) | 0.2013 | 1.2230 | 0.0787 | **0.011** |
| Number of T2 hyperintense lesions | -0.0171 | 0.9831 | 0.0106 | 0.107 |
| Number of T1 contrast-enhancing lesions | -0.0342 | 0.9663 | 0.1829 | 0.851 |
| κ-FLC index | 0.0197 | 1.0199 | 0.0053 | **<0.001** |
| sNfL Z score | 0.7575 | 2.1330 | 0.2573 | **0.003** |

Legend:

Disease duration was the time between symptom onset and lumbar puncture. Age was determined at the time of lumbar puncture. Number of MRI lesions were also determined at baseline. Follow-up duration was the time between disease onset and the last clinical visit.

*Abbreviations*: FLC, free light chain; MRI, magnetic resonance imaging; sNfL, serum neurofilament light

**Table e-4**: Cox regression analysis including type of disease manifestation to identify predictors for time to second clinical attack

|  | **Coefficient** | **Hazard ratio** | **Standard error** | **P value** |
| --- | --- | --- | --- | --- |
| Age (years) | -0.0969 | 0.9076 | 0.0483 | **0.045** |
| Sex (ref: male) | -0.1552 | 0.8562 | 0.7066 | 0.826 |
| Disease duration (days) | -0.0293 | 0.9711 | 0.0141 | **0.037** |
| Follow-up duration (months) | 0.2629 | 1.3007 | 0.1071 | **0.014** |
| Type of disease manifestation (ref: optic neuritis) |  |  |  |  |
| Myelitis | -0.2082 | 0.8121 | 0.7064 | 0.768 |
| Brainstem/ cerebellum | -1.0884 | 0.3367 | 0.8413 | 0.196 |
| Number of T2 hyperintense lesions | -0.0262 | 0.9742 | 0.0154 | 0.089 |
| Number of T1 contrast-enhancing lesions | 0.0190 | 1.0192 | 0.2615 | 0.942 |
| DMT administration | 0.6511 | 1.9176 | 0.8351 | 0.436 |
| κ-FLC index | 0.0264 | 1.0267 | 0.0074 | **<0.001** |
| sNfL Z score | 0.9752 | 2.6518 | 0.3341 | **0.004** |

Legend:

Disease duration was the time between symptom onset and lumbar puncture. Age was determined at the time of lumbar puncture. Number of MRI lesions were also determined at baseline. Follow-up duration was the time between disease onset and the last clinical visit. DMT administration was determined until occurrence of second clinical attack or end of follow-up, respectively.

*Abbreviations*: DMT, disease-modifying treatment; FLC, free light chain; MRI, magnetic resonance imaging; sNfL, serum neurofilament light

**Table e-5**: Cox regression analysis including prior corticosteroid treatment to identifiy predictors for time to second clinical attack

|  | **Coefficient** | **Hazard ratio** | **Standard error** | **P value** |
| --- | --- | --- | --- | --- |
| Age (years) | -0.0980 | 0.9066 | 0.04671 | **0.036** |
| Sex (ref: male) | -0.5048 | 0.6036 | 0.7706 | 0.512 |
| Disease duration (days) | -0.0266 | 0.9737 | 0.0122 | **0.029** |
| Follow-up duration (months) | 0.3183 | 1.3748 | 0.1242 | **0.010** |
| Number of T2 hyperintense lesions | -0.0363 | 0.9644 | 0.0233 | 0.119 |
| Number of T1 contrast-enhancing lesions | -0.0670 | 0.9352 | 0.2836 | 0.813 |
| DMT administration | 0.9965 | 2.7089 | 0.7818 | 0.202 |
| Corticosteroid treatment prior LP | 0.4804 | 1.6167 | 1.4848 | 0.746 |
| κ-FLC index : Corticosteroid treatment prior LP | -0.0122 | 0.9879 | 0.0140 | 0.383 |
| sNfL z score : Corticosteroid treatment prior LP | 0.6209 | 1.8605 | 0.6618 | 0.348 |
| κ-FLC index | 0.0314 | 1.0319 | 0.0090 | **<0.001** |
| sNfL Z score | 0.7807 | 2.1831 | 0.4195 | **0.063** |

Legend:

Disease duration was the time between symptom onset and lumbar puncture. Age was determined at the time of lumbar puncture. Number of MRI lesions were also determined at baseline. Follow-up duration was the time between disease onset and the last clinical visit. DMT administration was determined until occurrence of second clinical attack or end of follow-up, respectively.

*Abbreviations*: DMT, disease-modifying treatment; FLC, free light chain; LP, lumbar puncture; MRI, magnetic resonance imaging; sNfL, serum neurofilament light

**Table e-6**: Cox regression analysis including storage time to identifiy predictors for time to second clinical attack

|  | **Coefficient** | **Hazard ratio** | **Standard error** | **P value** |
| --- | --- | --- | --- | --- |
| Age (years) | -0.0533 | 0.9481 | 0.0326 | 0.102 |
| Sex (ref: male) | 0.4180 | 1.5189 | 0.6671 | 0.531 |
| Disease duration (days) | -0.0261 | 0.9742 | 0.0116 | **0.024** |
| Follow-up duration (months) | 0.2045 | 1.2269 | 0.0812 | **0.012** |
| Number of T2 hyperintense lesions | -0.0206 | 0.9796 | 0.0129 | 0.110 |
| Number of T1 contrast-enhancing lesions | -0.0887 | 0.9151 | 0.2173 | 0.683 |
| DMT administration | 0.5456 | 1.7256 | 0.7708 | 0.479 |
| Storage time (years) | 0.0613 | 1.0633 | 0.1129 | 0.587 |
| κ-FLC index | 0.0199 | 1.0201 | 0.0058 | **<0.001** |
| NfL Z score (pg/ml) | 0.7670 | 2.1534 | 0.2672 | **0.004** |

Legend:

Disease duration was the time between symptom onset and lumbar puncture. Age was determined at the time of lumbar puncture. Number of MRI lesions were also determined at baseline. Follow-up duration was the time between disease onset and the last clinical visit. DMT administration was determined until occurrence of second clinical attack or end of follow-up, respectively.

*Abbreviations*: CSF, cerebrospinal fluid; DMT, disease-modifying treatment; FLC, free light chain; MRI, magnetic resonance imaging; NfL, neurofilament light

**Table e-7**: **(A)** Cox regression analysis predicting time to second clinical attack considering demographics, clinical characteristics (without follow-up duration), κ-FLC index and sNfL Z score

|  | **Coefficient** | **Hazard ratio** | **Standard error** | **P value** |
| --- | --- | --- | --- | --- |
| Age (years) | -0.0281 | 0.9723 | 0.0276 | 0.3090 |
| Sex (ref: male) | 0.7906 | 2.2047 | 0.5671 | 0.1633 |
| Disease duration (days) | -0.0227 | 0.9776 | 0.0118 | 0.0554 |
| Number of T2 hyperintense lesions | -0.0089 | 0.9912 | 0.0115 | 0.4403 |
| Number of T1 contrast-enhancing lesions | -0.0537 | 0.9477 | 0.1965 | 0.7847 |
| DMT administration | 0.0982 | 1.1031 | 0.7515 | 0.8961 |
| κ-FLC index | 0.0181 | 1.0183 | 0.0050 | **0.0003** |
| NfL Z score (pg/ml) | 0.5795 | 1.7851 | 0.2327 | **0.0128** |

Legend:

Disease duration was the time between symptom onset and lumbar puncture. Age was determined at the time of lumbar puncture. Number of MRI lesions were also determined at baseline. DMT administration was determined until occurrence of second clinical attack or end of follow-up, respectively.

*Abbreviations*: DMT, disease-modifying treatment; FLC, free light chain; MRI, magnetic resonance imaging; sNfL, serum neurofilament light

**Table e-7**: **(B)** Relapse free probability at 12 months depending on κ-FLC index and sNfL Z score based on Cox regression analysis in Table e-7 (A)

|  | | **sNfL Z score** | | |
| --- | --- | --- | --- | --- |
|  |  | $\leq$**1.5** | $>$**1.5** $-$ **3** | $>$**3** |
| **κ-FLC index** | $\leq$**6.1** | **97%**  (92-100) | **90%**  (81-100) | **82%**  (69-99) |
|  | $>$**6.1** $-$ **100** | **95%**  (89-100) | **83%**  (72-96) | **72%**  (56-92) |
|  | $>$**100** | **66%**  (48-90) | **24%**  (16-35) | **8%**  (6-10) |

Legend:

The probability (and 95%-confidence interval) of staying relapse-free within 12 months after disease onset is given for each of the possible combinations of negative, elevated and highly elevated κ-FLC index and sNfL Z score.

*Abbreviations*: κ-FLC, κ free light chain; sNfL, serum neurofilament light

**Table e-8**: Cox regression analysis identifiying κ-FLC index and CSF NfL as predictors for time to second clinical attack

|  | **Coefficient** | **Hazard ratio** | **Standard error** | **P value** |
| --- | --- | --- | --- | --- |
| Age (years) | -0.0448 | 0.9562 | 0.0291 | 0.123 |
| Sex (ref: male) | 0.2466 | 1.2797 | 0.6162 | 0.689 |
| Disease duration (days) | -0.0285 | 0.9719 | 0.0143 | **0.047** |
| Follow-up duration (months) | 0.1980 | 1.2190 | 0.0860 | **0.021** |
| Number of T2 hyperintense lesions | 0.0005 | 1.0005 | 0.0185 | 0.979 |
| Number of T1 contrast-enhancing lesions | 0.0676 | 1.0700 | 0.1359 | 0.619 |
| DMT administration | -0.3718 | 0.6896 | 0.6389 | 0.561 |
| κ-FLC index | 0.0136 | 1.0137 | 0.0049 | **0.005** |
| CSF NfL (pg/ml) | 0.0002 | 1.0002 | 0.0001 | **0.042** |

Legend:

Disease duration was the time between symptom onset and lumbar puncture. Age was determined at the time of lumbar puncture. Number of MRI lesions were also determined at baseline. Follow-up duration was the time between disease onset and the last clinical visit. DMT administration was determined until occurrence of second clinical attack or end of follow-up, respectively.

*Abbreviations*: CSF, cerebrospinal fluid; DMT, disease-modifying treatment; FLC, free light chain; MRI, magnetic resonance imaging; NfL, neurofilament light
