# Supplementary figures and images for: Kappa free light chain and neurofilament light independently predict early multiple sclerosis disease activity—a cohort study
Source: eBioMedicine. 2023 Apr 20;91:104573. doi: 10.1016/j.ebiom.2023.104573 (PMC10148088; doi:10.1016/j.ebiom.2023.104573)

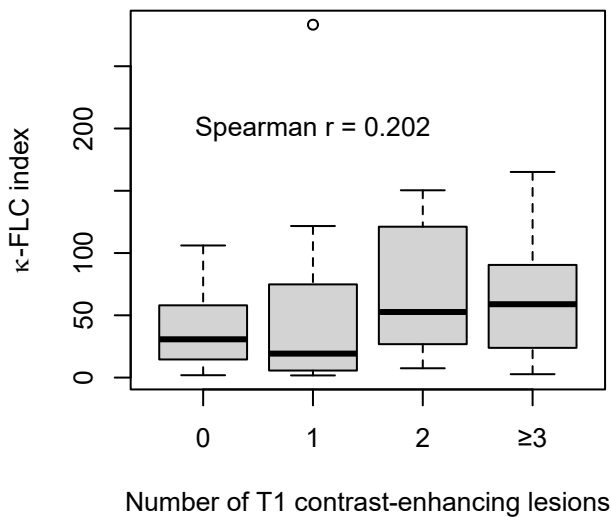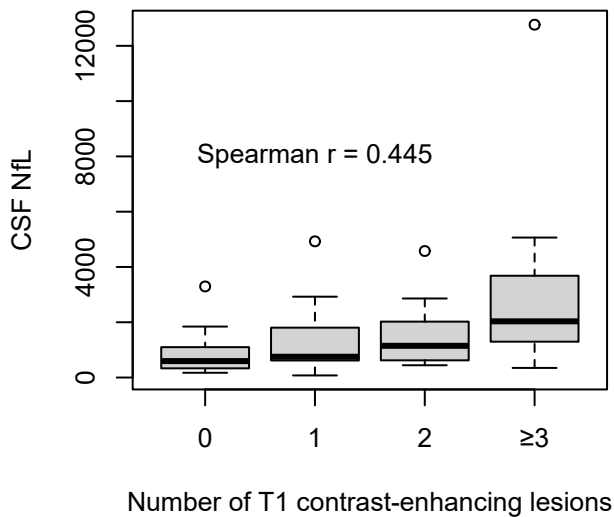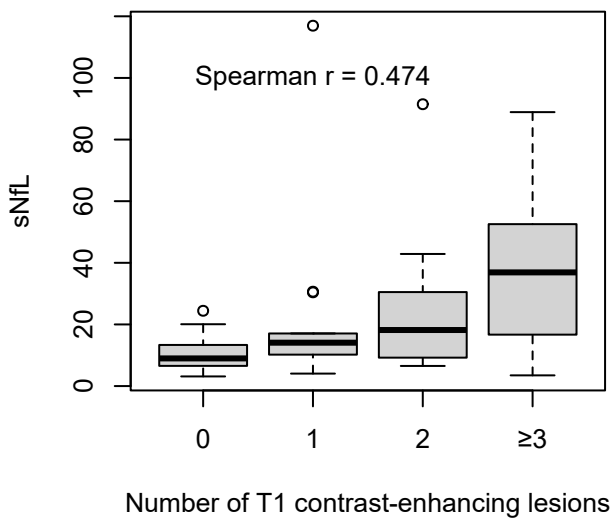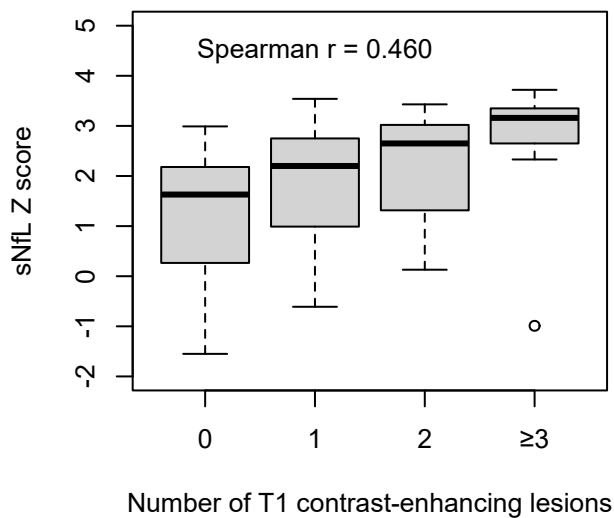

Supplement: Supplementary Fig. e-1 [file mmc2.pdf]

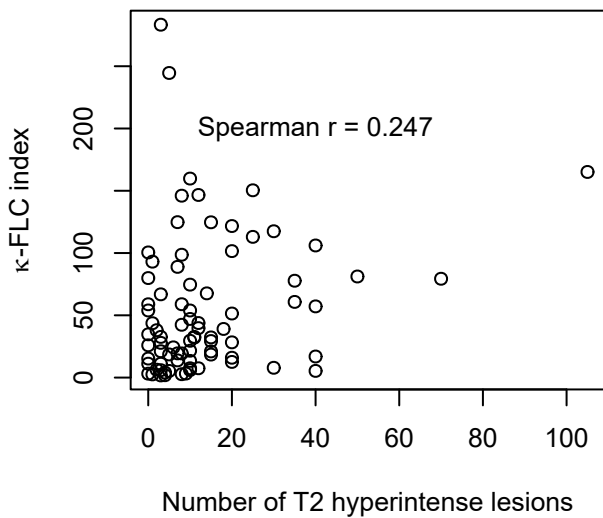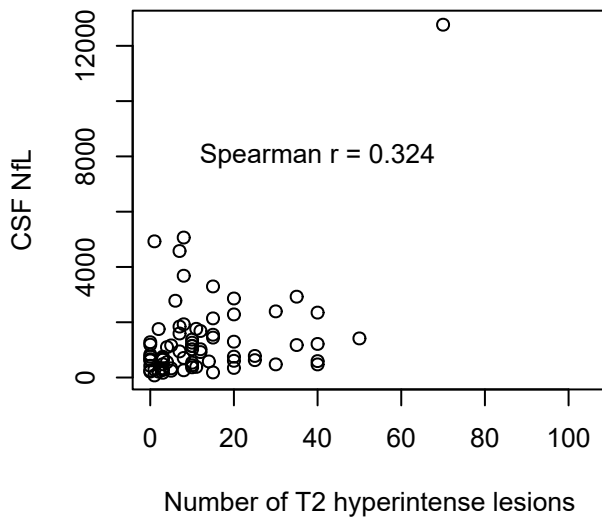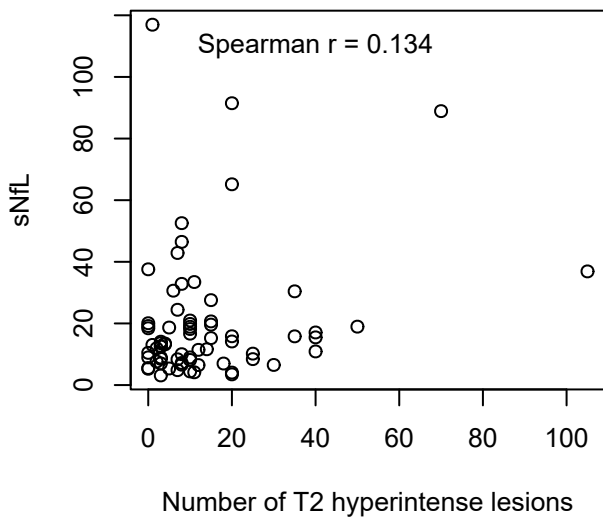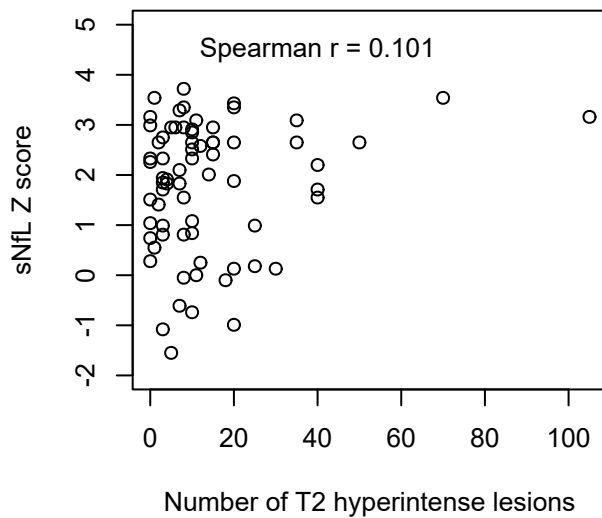

Supplement: Supplementary Fig. e-2 [file mmc3.pdf]

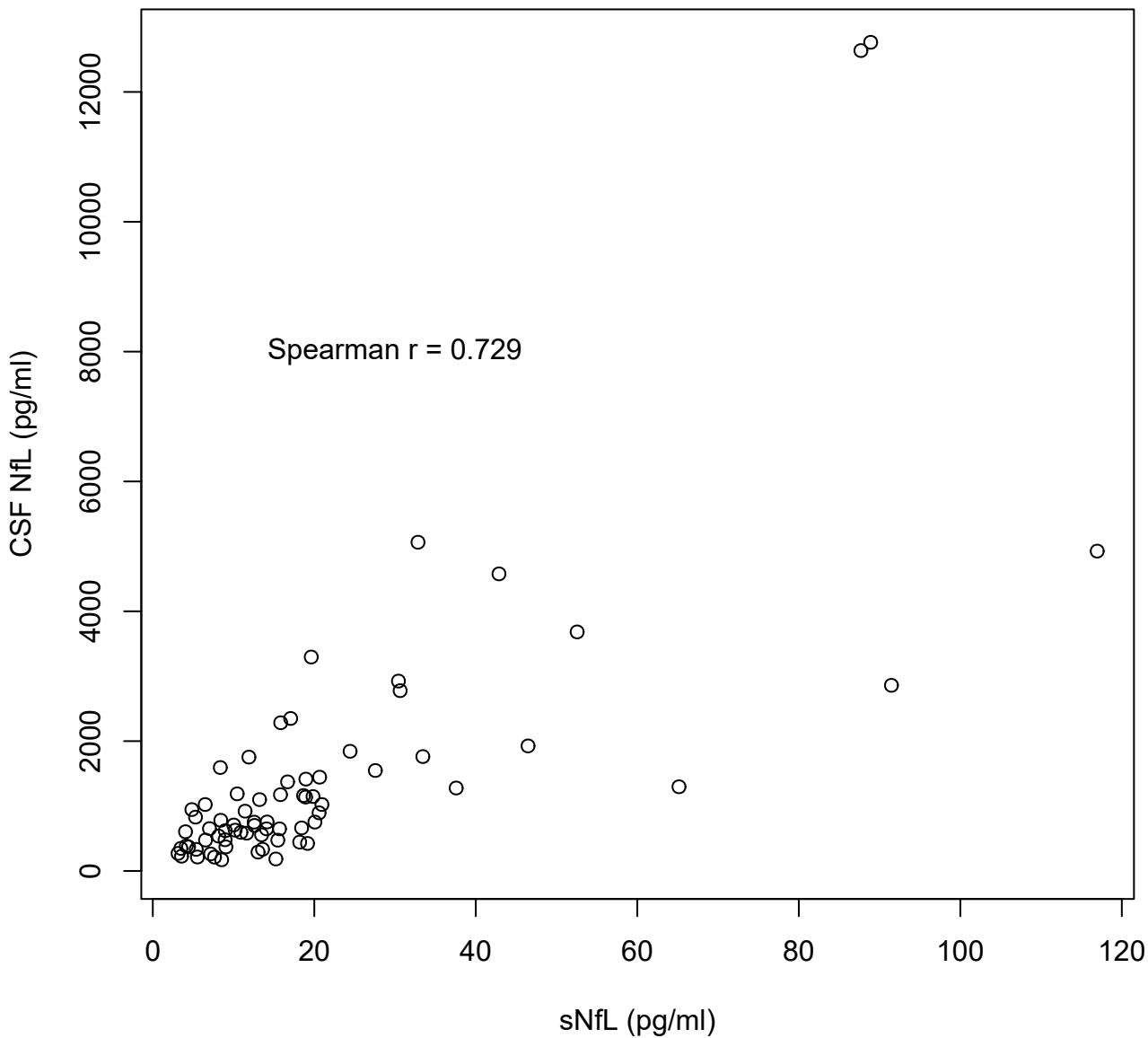

Supplement: Supplementary Fig. e-3 [file mmc4.pdf]

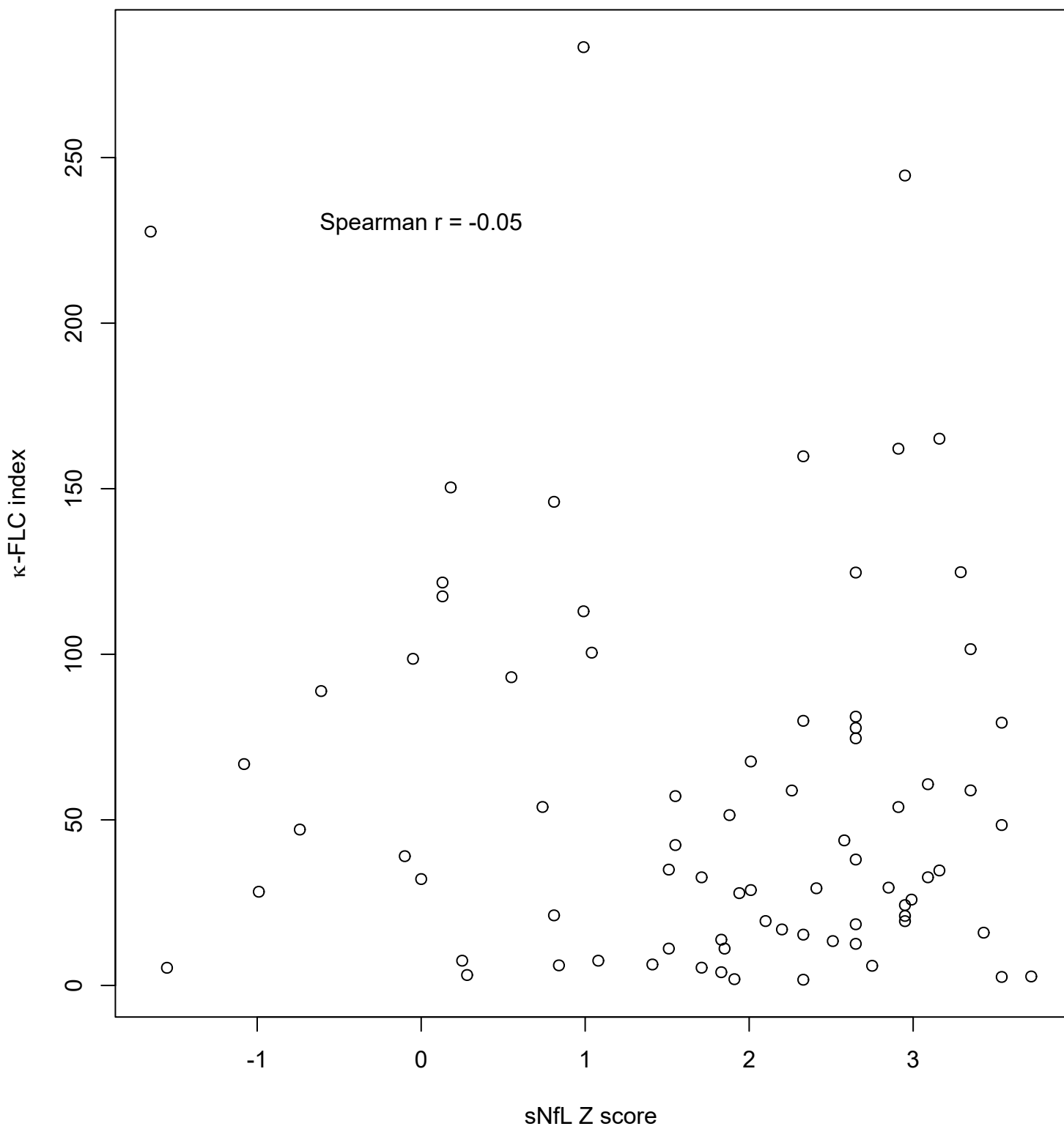

Supplement: Supplementary Fig. e-4 [file mmc5.pdf]

**A**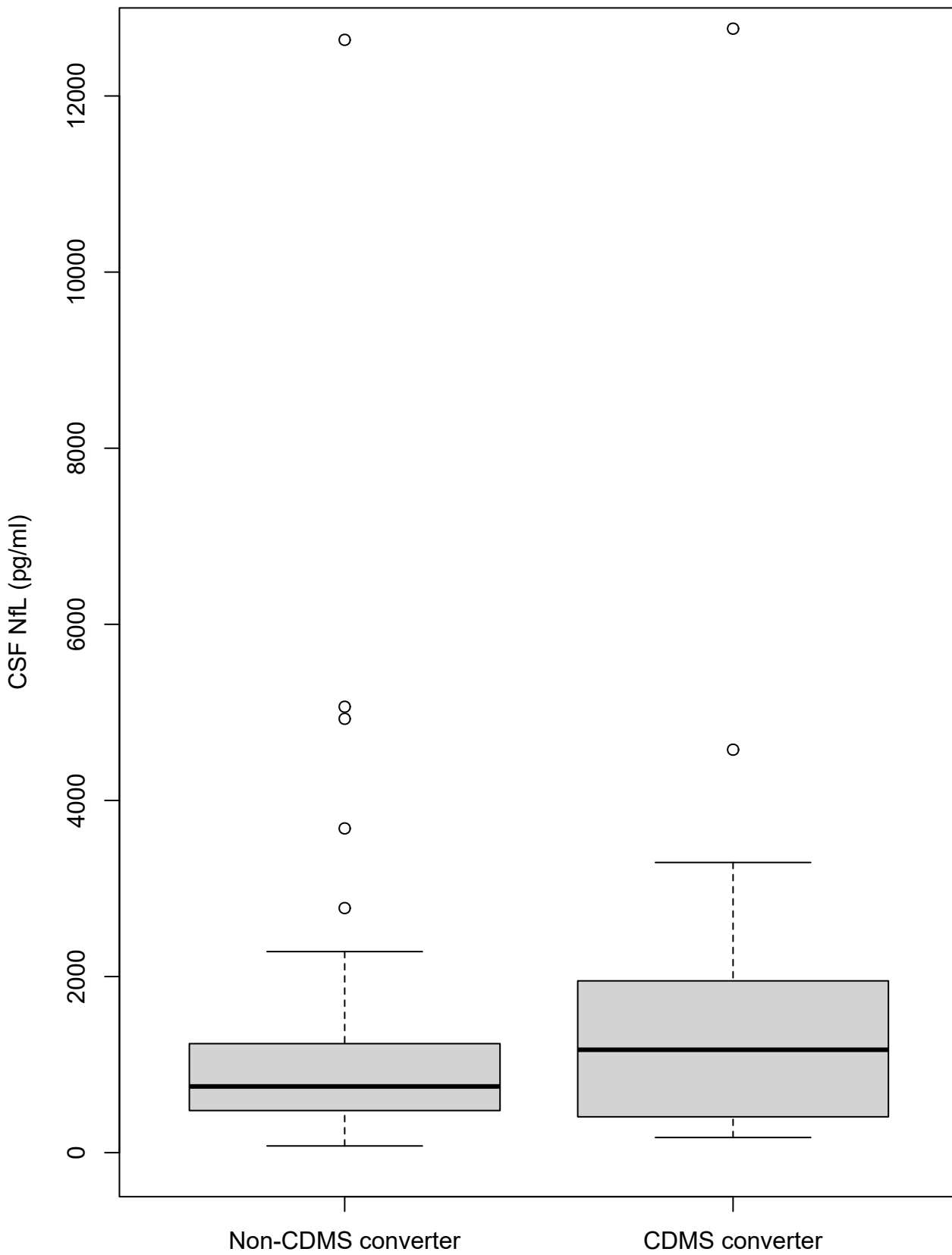**B**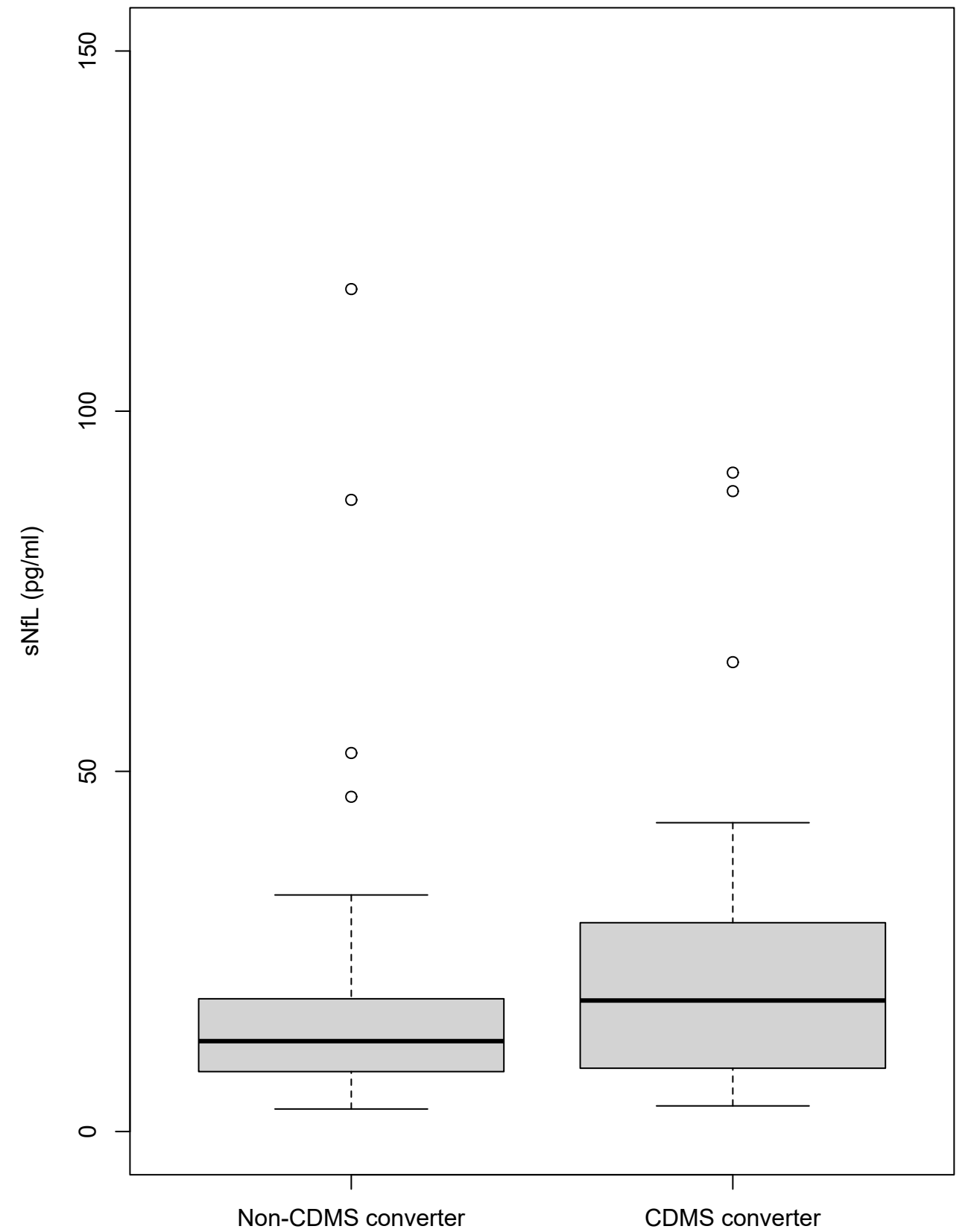

Supplement: Supplementary Fig. e-5 [file mmc6.pdf]

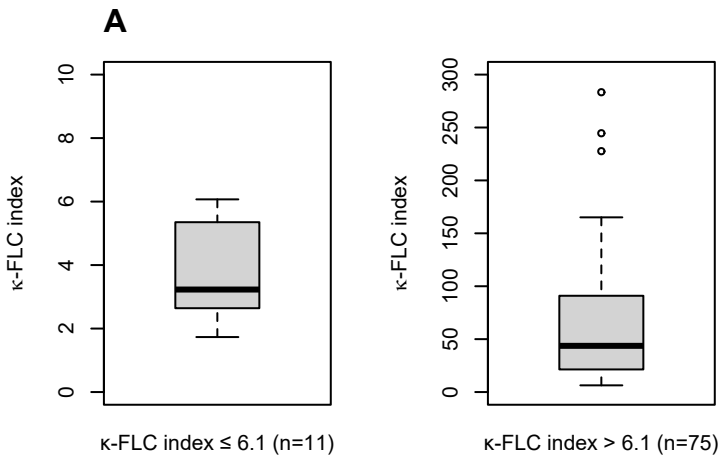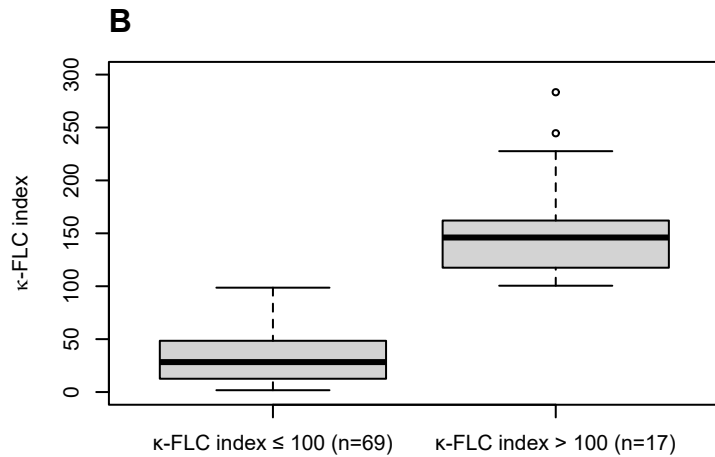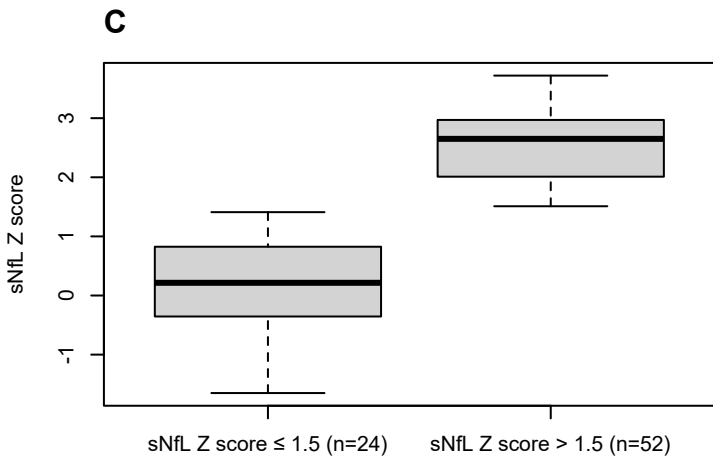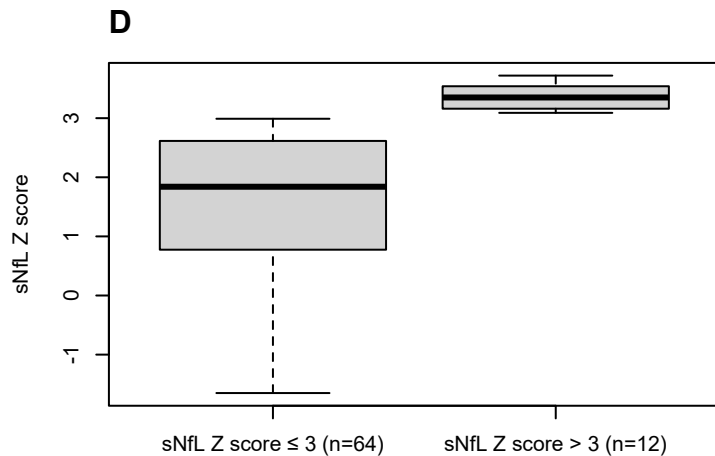

Supplement: Supplementary Fig. e-6 [file mmc7.pdf]

**Patients without prior corticosteroids (n=55)**

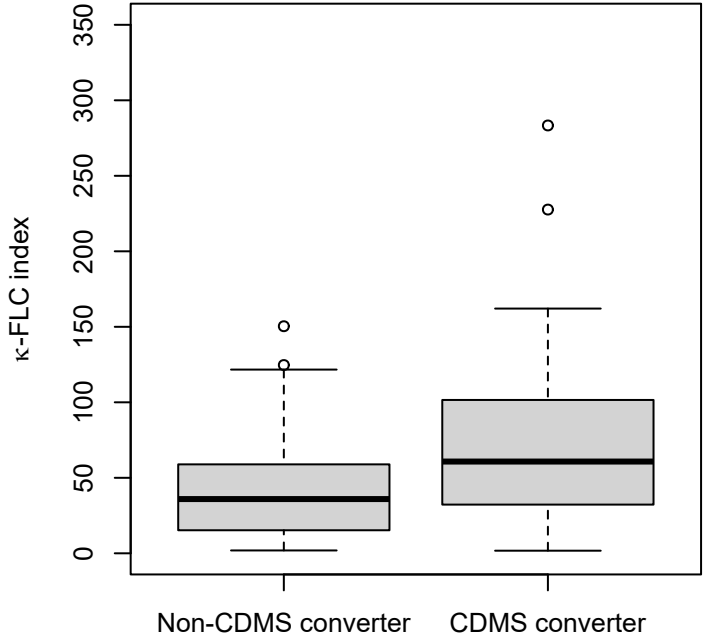

**All patients (n=86)**

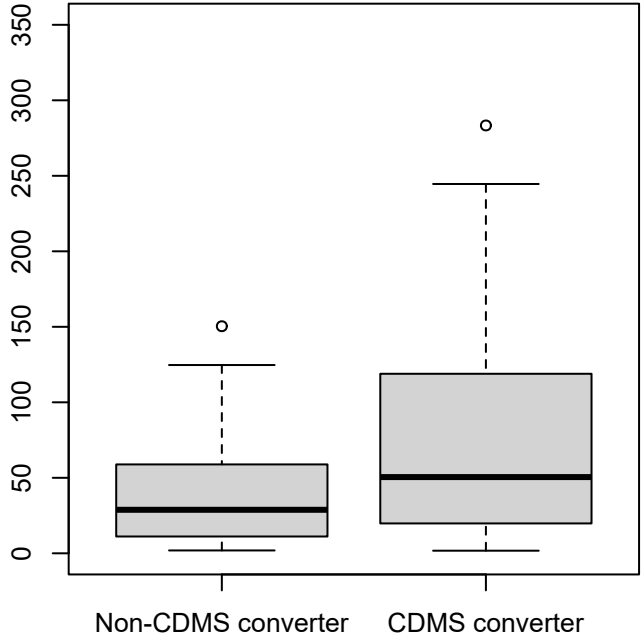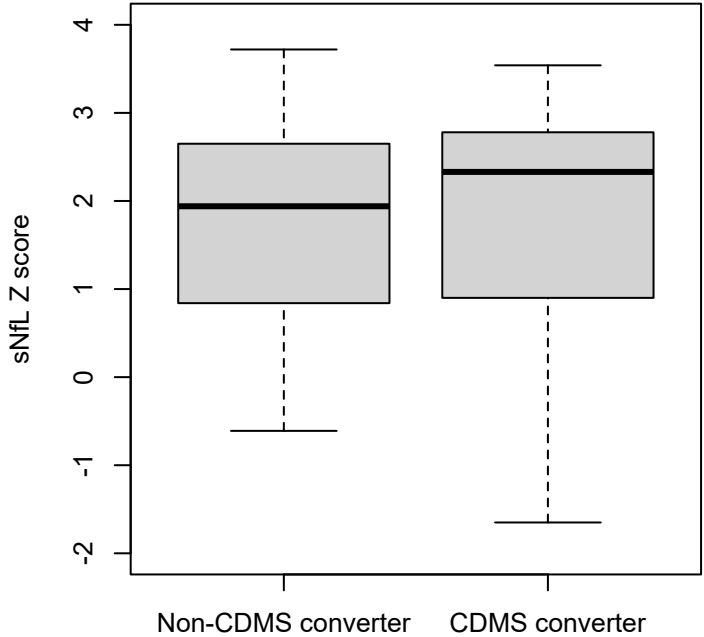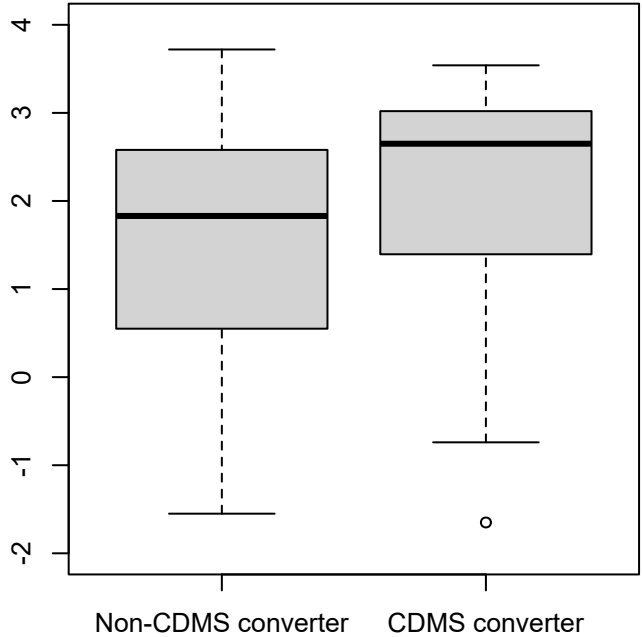

Supplement: Supplementary Fig. e-7 [file mmc8.pdf]

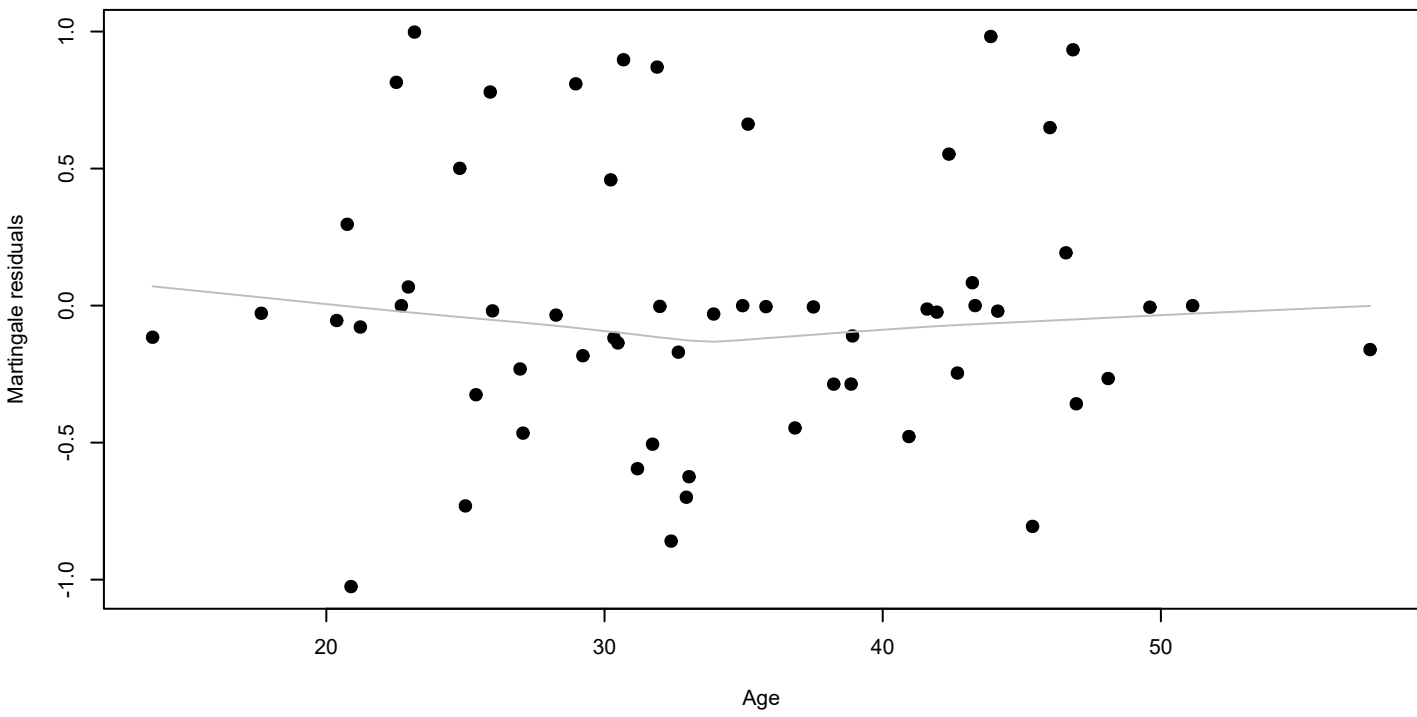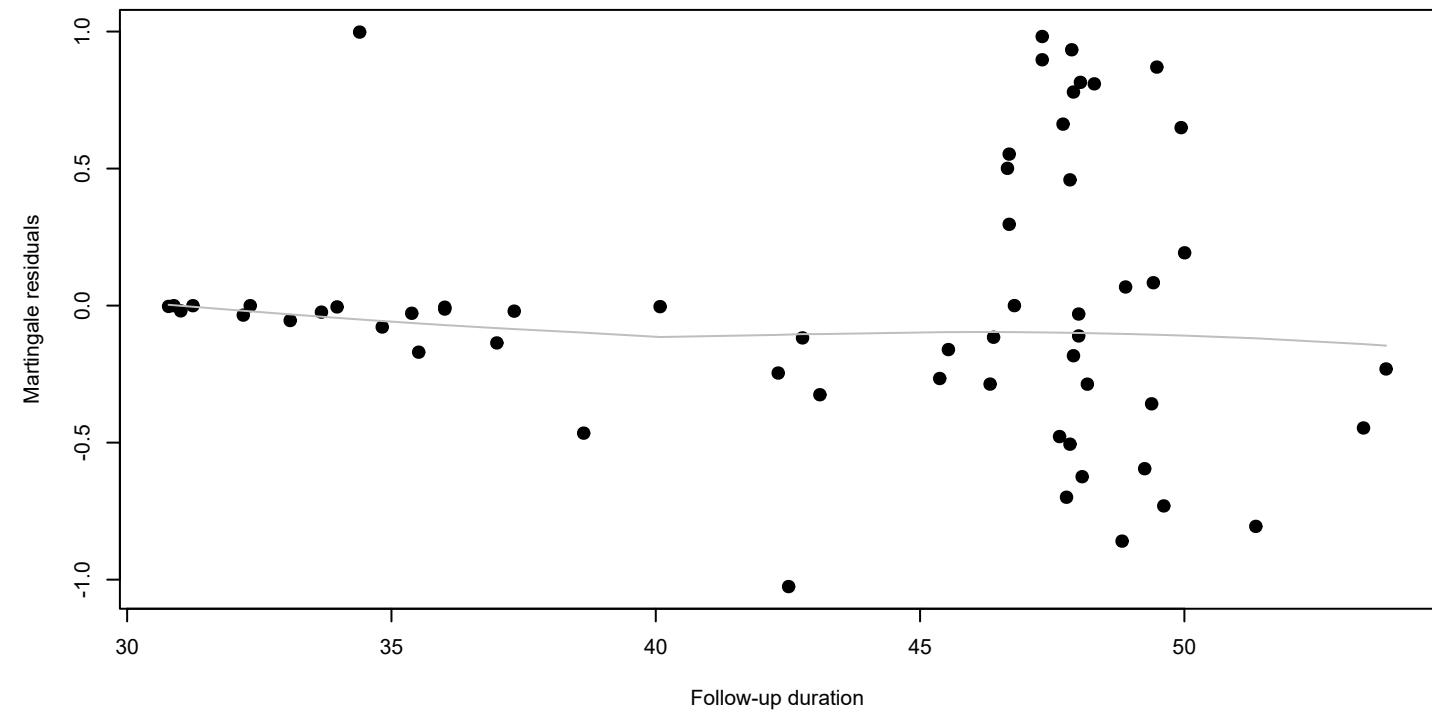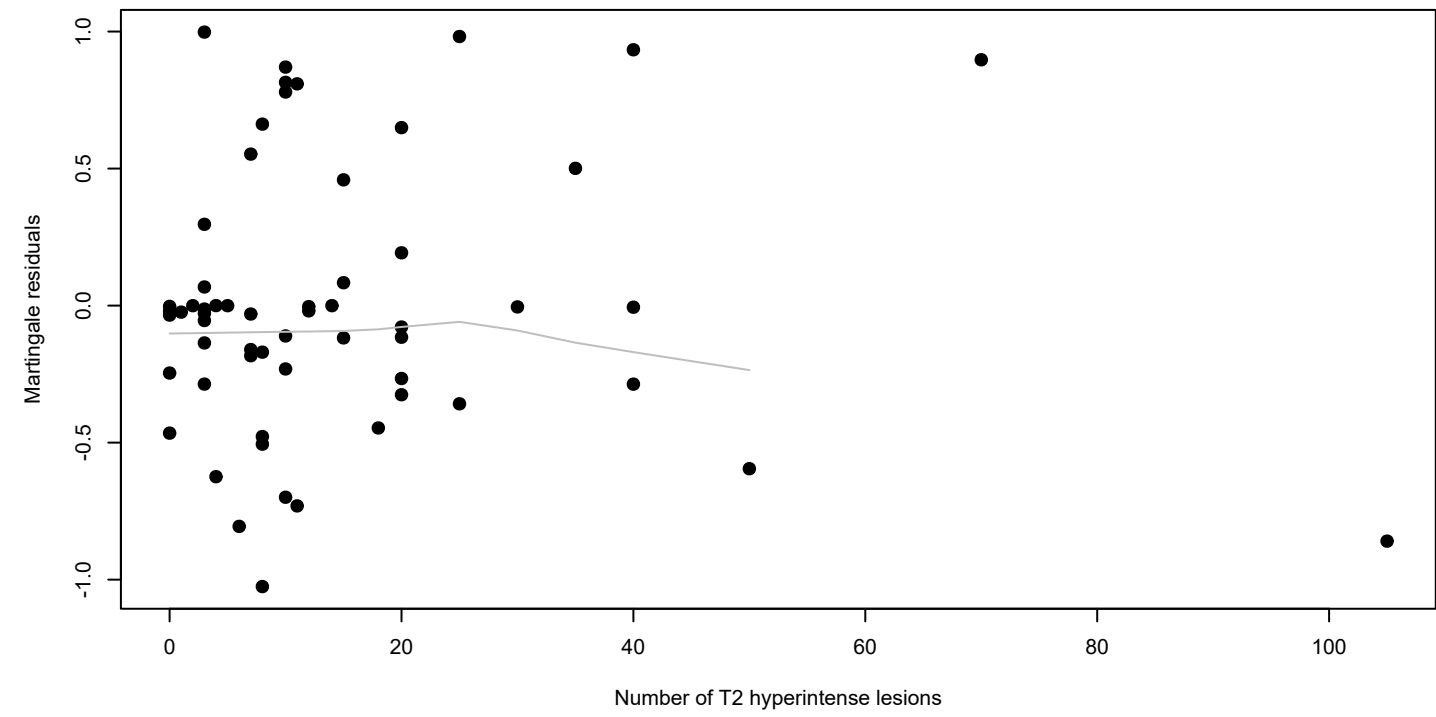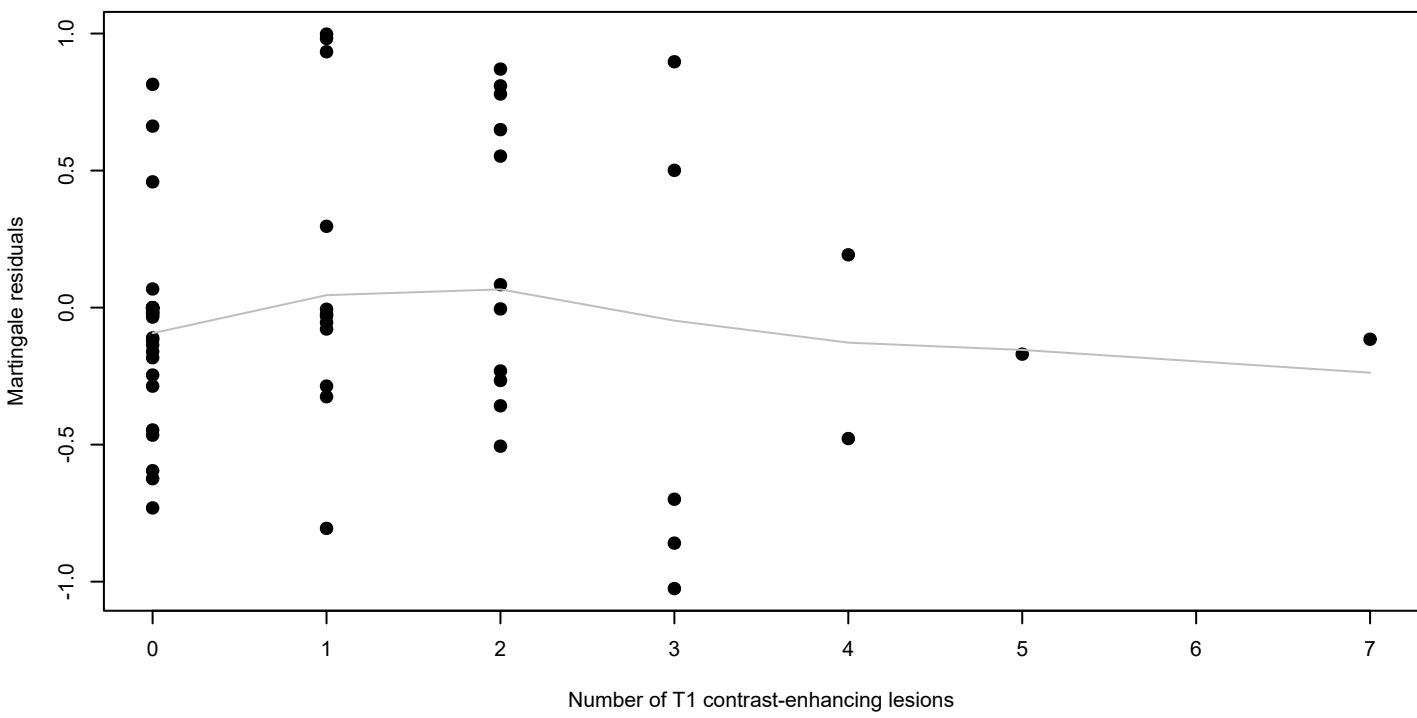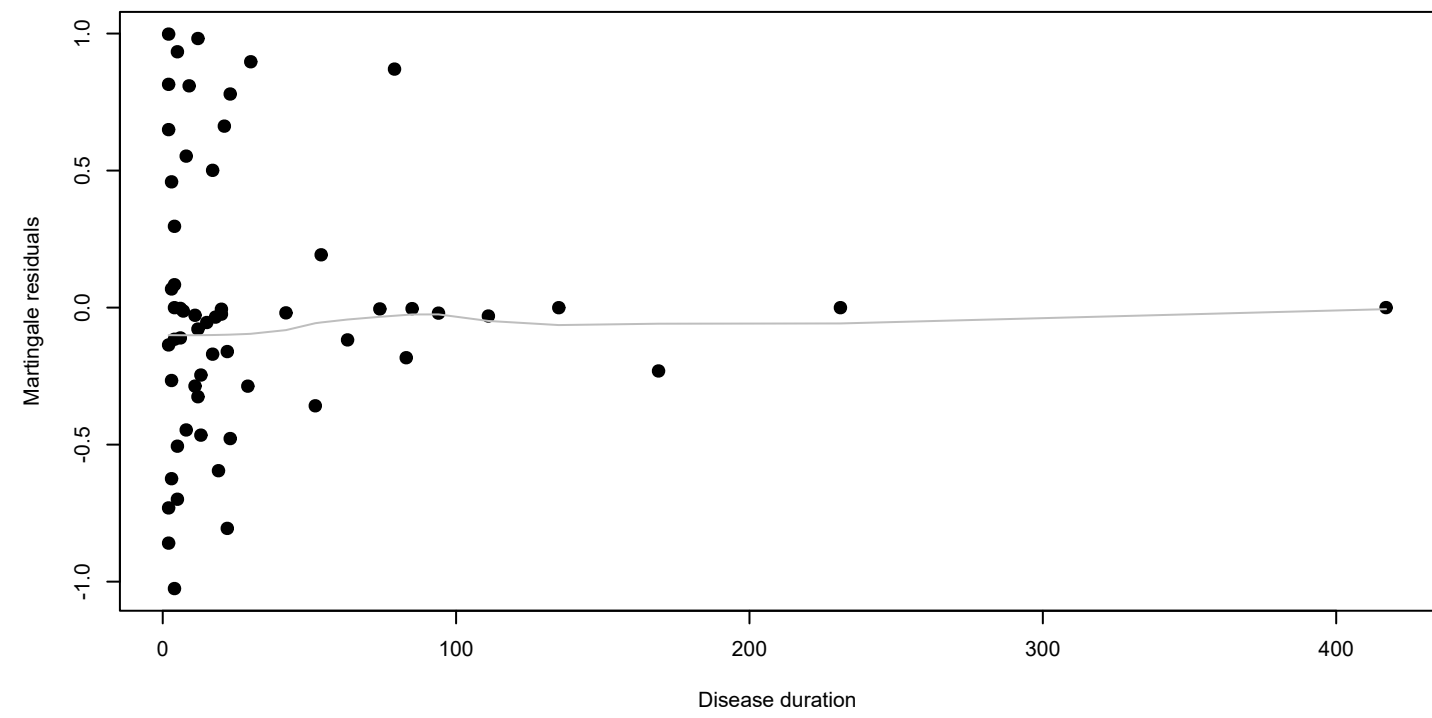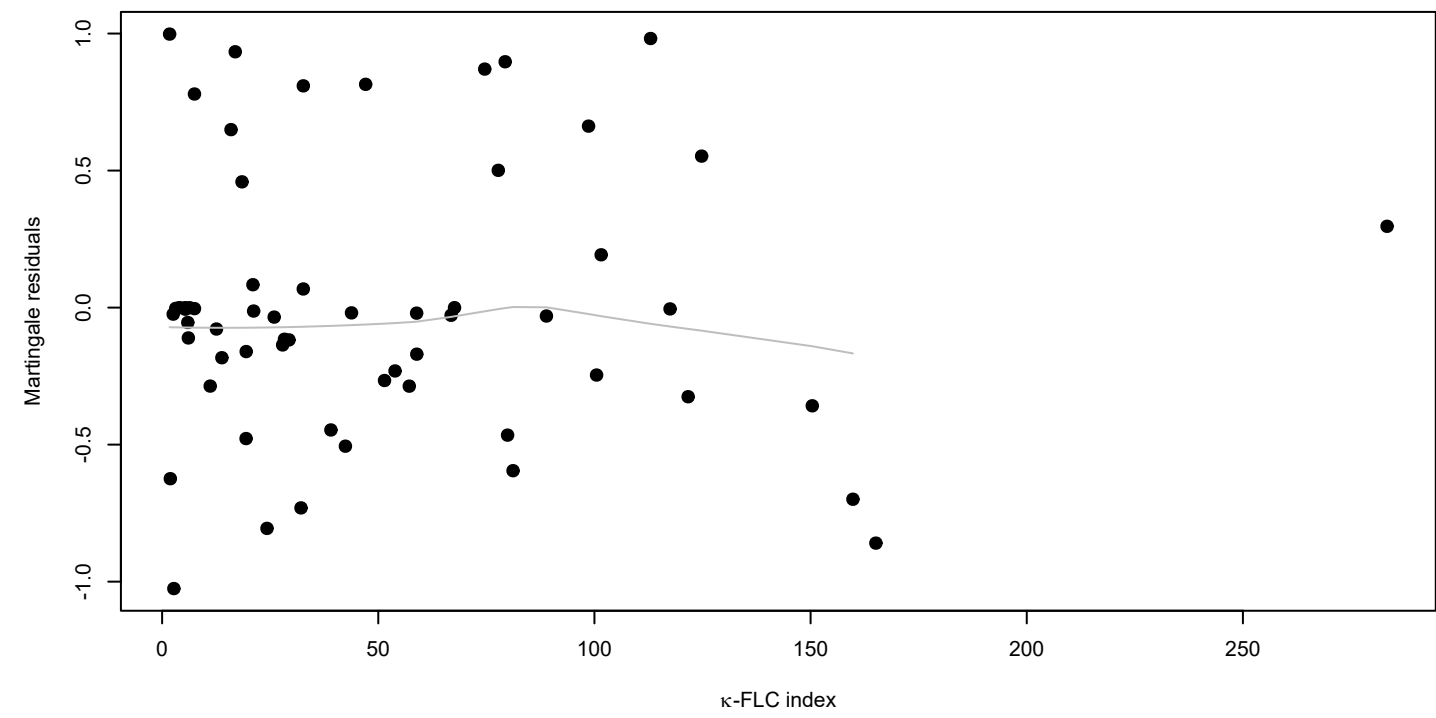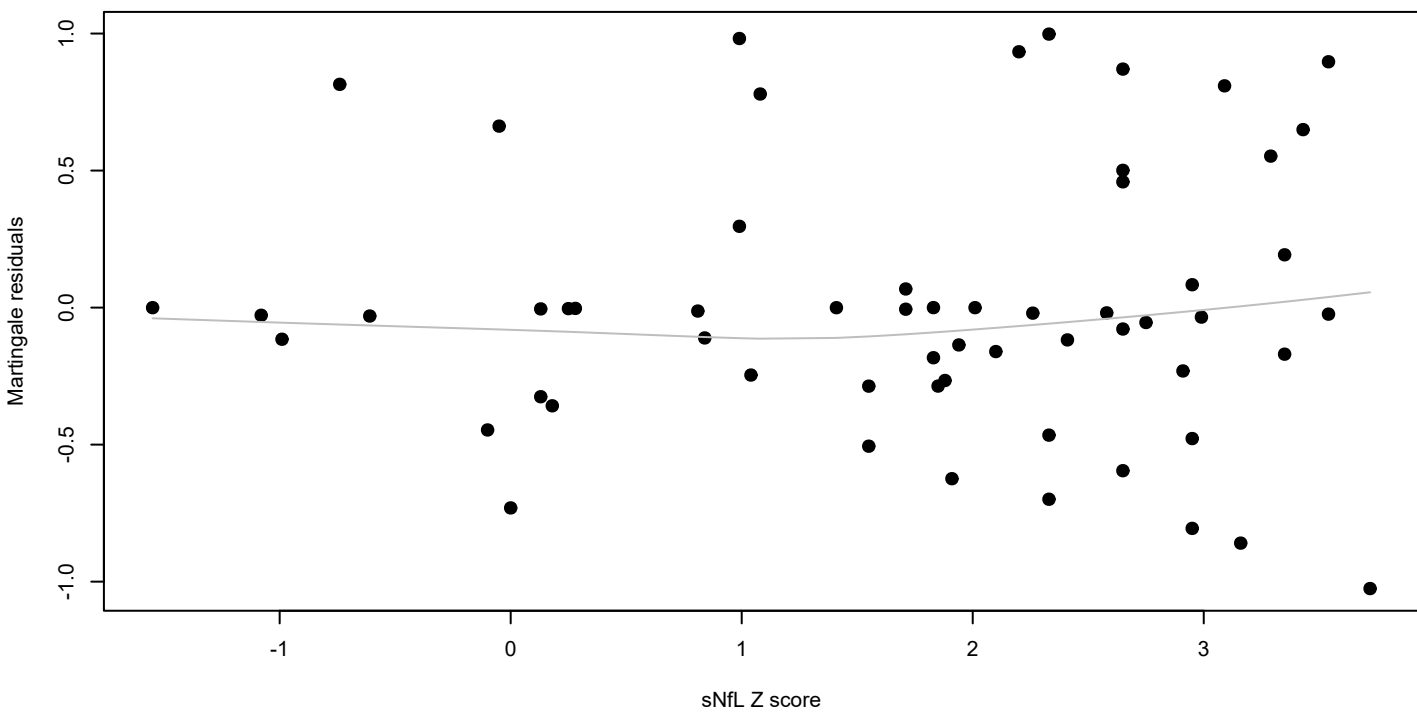

Supplement: Supplementary Fig. e-8 [file mmc9.pdf]
