# Supplementary material for: Kappa free light chain and neurofilament light independently predict early multiple sclerosis disease activity—a cohort study
Source: eBioMedicine. 2023 Apr 20;91:104573. doi: 10.1016/j.ebiom.2023.104573 (PMC10148088; doi:10.1016/j.ebiom.2023.104573)
Supplement: Supplementary figure captions [file mmc10.docx]

**Figure e-1**: Correlation of κ-FLC index, CSF NfL, sNfL and sNfL Z score with number of contrast-enhancing lesions on T1-weighted MRI

Legend:

*Abbreviations*: CE, contrast-enhancing lesions, CSF, cerebrospinal fluid; κ-FLC, κ free light chain; sNfL, serum neurofilament light

**Figure e-2**: Correlation of κ-FLC index, CSF NfL, sNfL and sNFL Z score with number of hyperintense lesions on T2-weighted MRI

Legend:

*Abbreviations*: CSF, cerebrospinal fluid; κ-FLC, κ free light chain; sNfL, serum neurofilament light

**Figure e-3**: Correlation of CSF NfL and sNfL concentration

Legend:

*Abbreviations*: CSF NfL, cerebrospinal fluid neurofilament light; sNfL, serum neurofilament light

**Figure e-4**: Correlation of κ-FLC index and sNfL Z score

Legend:

*Abbreviations*: sNfL, serum neurofilament light; κ-FLC, κ free light chain

**Figure e-5**: CSF and serum NfL concentrations in patients who converted to CDMS and in non-converters

Legend:

**(A)** CSF NfL concentration at baseline is higher in patients who convert to CDMS during 4-year follow-up compared to patients who remain relapse-free.

**(B)** Serum NfL concentration at baseline is higher in pateints who convert to CDMS during 4-year follow-up compared to patients who remain relapse-free.

*Abbreviations*: CDMS, clinically definite MS; CSF NfL, cerebrospinal fluid neurofilament light; sNfL, serum neurofilament light

**Figure e-6**: Distribution of κ-FLC index and sNfL Z score within low/ high categories

Legend:

*Abbreviations*: κ-FLC, κ free light chain; sNfL, serum neurofilament light

**Figure e-7**: κ-FLC index and sNfL Z in patients who converted to CDMS and in non-converters depending on baseline corticosteroid treatment

Legend:

*Abbreviations*: CDMS, clinically definite multiple sclerosis; κ-FLC, κ free light chain; sNfL, serum neurofilament light

**Figure e-8**: Martingale residuals dependent on the continuous covariates of the Cox regression

Legend:

The grey line shows the LOWESS smoother which uses locally-weighted polynomial regression (Cleveland 1981. LOWESS: A program for smoothing scatterplots by robust locally weighted regression. The American Statistician; 35:54 [doi:10.2307/2683591]).

Note: With sparse observations at the upper end, the functional form was no longer estimated via the LOWESS smoother due to overfitting.

*Abbreviations*: κ-FLC, κ free light chain; sNfL, serum neurofilament light
